# Prognostic and Predictive Value of the Clearseq1–4 Tumor Microenvironment Classification in Localized and Metastatic Clear-Cell Renal Cell Carcinoma

**DOI:** 10.1158/2767-9764.CRC-25-0548

**Published:** 2026-04-20

**Authors:** Lisa Kinget, Edward Scott McTaggart, Octavie Demeulenaere, Eduard Roussel, Bram Boeckx, Jessica Zucman-Rossi, Gabrielle Couchy, Henri Vandermeulen, Liesbeth De Wever, Marcella Baldewijns, Agnieszka Wozniak, Steven Joniau, Diether Lambrechts, Stefan Naulaerts, Abhishek D. Garg, Annelies Verbiest, Maarten Albersen, Benoit Beuselinck

**Affiliations:** 1 Department of General Medical Oncology, Leuven Cancer Institute, University Hospitals Leuven, Leuven, Belgium.; 2 Laboratory of Experimental Oncology (LEO), Department of Oncology, Leuven Cancer Institute, KU Leuven, Leuven, Belgium.; 3 Laboratory of Computational Oncology, Department of Oncology, KU Leuven, Leuven, Belgium.; 4 Laboratory of Cell Stress & Immunity (CSI), Department of Cellular and Molecular Medicine, KU Leuven, Leuven, Belgium.; 5 Department of Urology, University Hospitals Leuven, Leuven, Belgium.; 6 Laboratory of Translational Genetics, Department of Human Genetics, KU Leuven, Leuven, Belgium.; 7 VIB Center for Cancer Biology, VIB, Leuven, Belgium.; 8 Inserm, UMR-1138, Génomique fonctionnelle des tumeurs solides, Institut de recherche des Cordeliers, Université Paris Descartes, Paris, France.; 9 Department of Radiology, University Hospitals Leuven, Leuven, Belgium.; 10 Department of Pathology, University Hospitals Leuven, Leuven, Belgium.; 11 Department of Medical Oncology, Multidisciplinary Oncological Center Antwerp (MOCA), University Hospital Antwerp, Edegem, Belgium.

## Abstract

The ccrcc1–4 transcriptomic subtypes were previously identified in fresh-frozen clear-cell renal cell carcinoma (ccRCC) samples and have proven their prognostic value after nephrectomy/metastasectomy and predictive value for first-line vascular endothelial growth factor receptor–tyrosine kinase inhibitors (VEGFR-TKI). We aimed to create a consensus molecular classification approach called Clearseq1–4 on formalin-fixed, paraffin-embedded (FFPE) tissues and to evaluate its prognostic and predictive value across the RCC treatment landscape. RNA sequencing of tumoral FFPE tissue was performed. A classifier called Clearseq was designed to determine ccrcc1–4 subtypes. Subtypes were correlated with outcomes after surgical and systemic therapies. External validation and characterization at the single-cell transcriptomic level were pursued. A total of 668 tumoral samples (337 primary tumors and 331 metastases) from 364 patients were assigned to ccrcc1, ccrcc2, ccrcc3, and ccrcc4 tumors. The angiogenic ccrcc2 subtype had a favorable prognosis after nephrectomy in a localized setting, after cytoreductive nephrectomy, and upon metastasectomy with curative intent and was correlated with improved outcomes on first-line VEGFR-TKIs. Ccrcc4 tumors were enriched for sarcomatoid features and had the largest treatment benefit from immune checkpoint blockade (ICB) treatment in any line, resulting in overall survival outcomes comparable with those of less aggressive subtypes. These findings were corroborated in a post-nephrectomy cohort and external cohorts of metastatic patients treated with ICB and/or angiogenesis inhibitors. We created a consensus molecular classification approach, called Clearseq1–4, in order to predict the ccrcc1–4 molecular subtype on FFPE tissues and confirmed its performance with respect to previous biomarker findings for both surgical and systemic treatment approaches.

**Significance:**

Clear-cell kidney cancers display a more indolent or aggressive clinical behavior after surgery and different sensitivities to currently available medical therapies: immune therapy or angiogenesis inhibitors. We developed an easy-to-use molecular classification that divides these tumors into four subgroups predicting outcomes after surgery or upon medical therapy.

## Introduction

Clear-cell renal cell carcinoma (ccRCC) accounts for more than 80% of all kidney cancer cases and the majority of its cancer-related mortality (1). Despite the range of traditional clinical and histologic parameters available (2), patient heterogeneity in disease aggressiveness and response to systemic therapies varies strongly between patients and is not adequately captured by existing classification modalities (2). Advances in research technologies have increasingly corroborated this remarkable heterogeneity within ccRCC. In particular, at the transcriptomic level, the existence of several subtypes has been demonstrated by independent research groups. Initially, two distinct clusters, named ccA and ccB, were discovered by Brannon and colleagues (3), with the two clusters differing in disease-free survival (DFS) after nephrectomy. The ClearCode34 classifier was later developed to determine ccA and ccB (4). Subsequent research delineated four molecular subgroups (i.e., ccrcc1–4; ref. 5), which showed predictive value for angiogenesis inhibitors as well as prognostic value after nephrectomy and metastasectomy with curative intent (MWCI; refs. 6, 7). In parallel, the existence of four comparable subtypes (m1–m4) was found independently in both the TCGA-Kidney Renal Cell Carcinoma (KIRC) cohort (8) and the COMPARZ trial cohort (9). Finally, a large translational effort on the IMmotion151 trial observed seven distinct molecular subsets (10) that strongly overlap with the ccrcc1–4 subtypes. More specifically, ccrcc2 corresponds to the angio-stromal (cluster 1)/angio (cluster 2) subtypes, whereas ccrcc4 shares features of the T-effector/proliferative (cluster 4) subtype. Ccrcc1 demonstrates properties of the complement/Ω-oxidation (cluster 3), proliferative (cluster 5), and stromal/proliferative (cluster 6) subtypes. Within the IMmotion151 trial, these showed differential responses to atezolizumab–bevacizumab versus sunitinib. This classification was later validated in the Javelin Renal 101 cohort (avelumab–axitinib vs. sunitinib) and CheckMate025 cohort (nivolumab vs. everolimus; refs. 11, 12).

Given the differences in tumor microenvironment (TME) and response to systemic therapy observed in these translational efforts, it was hypothesized that these subtypes could support first-line treatment decision-making. Subgroups with increased angiogenesis showed enhanced susceptibility to antiangiogenic treatments such as vascular endothelial growth factor–tyrosine kinase inhibitors (VEGFR-TKI) in retrospective series (5). Meanwhile, two biomarker-driven trials were designed to prospectively investigate if the more aggressive, inflamed subtypes showed a better response to immune checkpoint blockade (ICB). The BIONIKK trial randomized patients with subtype ccrcc2/3 between ipilimumab/nivolumab and sunitinib and those with subtypes ccrcc1/4 between ipilimumab/nivolumab and nivolumab (13). Herein, the authors observed that the proangiogenic subtype ccrcc2 had a high objective response rate (ORR) with both treatments but had a longer median progression-free survival (mPFS) on VEGFR-TKI compared with ipilimumab–nivolumab. In the ccrcc4 subtypes, comparably high response rates (RR) and PFS were observed with both nivolumab and ipilimumab–nivolumab, whereas the ccrcc1 subtype seemed to benefit from the addition of ipilimumab to nivolumab (13). Similarly, the phase II OPTIC trial is ongoing, but no results have been reported yet (14).

Despite having different numbers of clusters, certain overarching subgroups are consistently identified in all studies. The first large subgroup contains the angiogenesis-enriched tumors [ccA in ClearCode34, m1 cluster in TCGA-KIRC, ccrcc2 in ccrcc1–4, cluster 3 in Hakimi and colleagues, and the Angio (2) and Angio/stromal (1) subset in IMmotion151]. These tumors showed strong downregulation of cell-cycle pathways, with patients having a less aggressive disease and a favorable response to angiogenesis inhibition (3, 5, 8, 9). The remaining tumors, corresponding to ccB in the ClearCode34 classification, have downregulated angiogenesis and higher cell-cycle expression in a largely exclusive manner. Among these tumors, other classifications identified a subgroup with strong T-cell infiltration [ccrcc4, IMmotion151 T-effector/proliferative subtype (cluster 4)], with generally aggressive tumor behavior, poor response to VEGFR-TKIs, and increased benefit from ICB. The remaining tumors without such characteristic T-cell infiltration generally have strong proliferation and myeloid infiltration [ccrcc1, IMmotion151 cluster 5 (proliferative), cluster 6 (proliferative/stromal), and cluster 3 (complement/Ω oxidation); refs. 3, 5, 8, 9]. For these groups, findings are highly consistent in terms of transcriptomic, genomic, clinicopathologic, and biomarker value characteristics, which is remarkable, given that they were derived from several distinct cohorts spanning thousands of patients with ccRCC across different continents and disease stages.

Despite the consistencies across studies, the determination and validation of these molecular subtypes across different cohorts remain challenging due to the need to reconstruct the class labels reported in the clinical trials. Second, the value of these subtypes for current first-line therapies, in particular for dual ICB (ipilimumab-nivolumab), has not been cross-connected to single-cell behaviors.

In this study, we aimed to create a consensus molecular classification approach, called Clearseq1–4, including hallmark features (Supplementary Table S1) from previously published subgroup studies to assign, with a classification algorithm, the ccrcc1–4 subgroup labels to bulk gene expression profiles from formalin-fixed, paraffin-embedded (FFPE) samples (3, 5, 8, 9). We subsequently evaluated the clinicopathologic characteristics among subgroups as well as their impact on treatment outcomes in surgical and systemic therapies. Moreover, we studied the characteristics of the signatures underlying the classifier at single-cell resolution. Finally, we pursued external validation in TCGA-KIRC as well as the Javelin Renal 101 and IMmotion150 cohorts.

## Materials and Methods

### Clinical cohort

**Figure 1.**
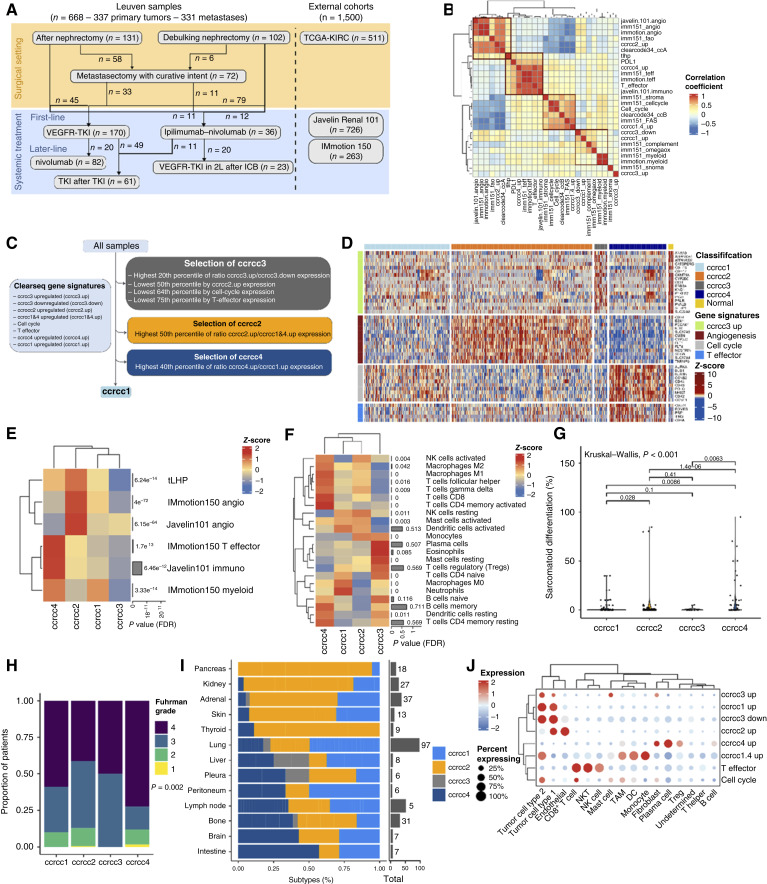
Transcriptomic and clinical characteristics of the Clearseq molecular subtypes at bulk and single-cell resolution. **A,** Flowchart showing patient distribution across different clinical contexts. **B,** Heatmap of Spearman correlation coefficients of the correlation matrix. Rows and columns clustered by the WARD.D method. **C,** Flowchart describing classification. **D,** Heatmap showing expression of key gene signatures across subtypes. **E,** Distribution of state-of-the-art RCC biomarker signatures across subtypes. *P* values from the Kruskal–Wallis test, FDR-corrected. Row-normalized, WARD.D clustering. **F,** Distribution of CIBERSORTx signatures across subtypes. *P* values from Kruskal–Wallis test, FDR-corrected. **G,** Percentage of sarcomatoid differentiation across subtypes. **H,** Fuhrman tumor grade across subtypes. **I,** Relative distribution of molecular subtype per metastatic site. **J,** Dot plot showing expression of the Clearseq signatures across cell types in a single-cell dataset (Bi and colleagues; ref. 24).

A cohort of 668 samples (337 primary kidney tumors and 331 metastases) with ccRCC was retrospectively collected at University Hospital Leuven, Belgium, in various disease settings: patients with localized disease who underwent nephrectomy, patients who underwent MWCI, and patients in a metastatic setting who underwent debulking nephrectomy. Additionally, we included patients treated with systemic treatment with VEGFR-TKI in the first line, VEGFR-TKI after ICB, and ICB in later lines and in the first line (Fig. 1A; Supplementary Tables S2–S12). Patients with non–clear-cell histology were excluded. Clear-cell histology, Fuhrman grade, Leibovich score, and sarcomatoid differentiation were confirmed by an expert genitourinary pathologist (M. Baldewijns). Ethical approval was obtained from the local ethical committee (approval number S53479/S63833). Written informed consent was obtained from all patients who were still alive. The hospital policy allows the use of biological material from deceased patients, except if they signed an opt-out document. The study was conducted in accordance with the Declaration of Helsinki. The best overall response was evaluated using RECIST version 1.1 criteria (for VEGFR-TKI treatment) and iRECIST criteria (for ICB treatment). All outcome correlations (after nephrectomy in the curative setting, after debulking nephrectomy, after metastasectomy, and during systemic therapy with VEGFR-TKIs and ICBs) were performed with molecular data from primary kidney tumors. Molecular data from metastases were only used for the study of the prevalence of molecular subtypes in distinct host organs. Only a reduced number of patients had both primary tumors and metastases available. In this study, we did not focus on the heterogeneity between primary tumors and metastases and its impact on outcomes, as this has been the topic of a previous publication (15).

### RNA extraction and sequencing

The FFPE tissues were archived in a specialized storage facility with controlled access and at room temperature. After RNA extraction from FFPE tissues of the primary tumor, next-generation sequencing was performed as previously reported (15). RNA was extracted using Maxwell RSC RNA FFPE kits (cat. #AS1440) before storage at −80°C. cDNA library preparation was performed using the Forward QuantSeq 3′ mRNA-Seq Library Prep Kit for Illumina (Lexogen), and clonal clusters were produced using Illumina cBOT before sequencing with the HiSeq 400 kit.

Samples sequenced before October 2020 were aligned to the human reference genome hg19; all later samples were aligned to hg38. All alignments were performed using HiSat2 (RRID:SCR_015530). Alignments to hg19 were quantified using featureCounts (RRID:SCR_012919), and alignments to hg38 were quantified using htseq-count (RRID:SCR_011867). Genes with counts per million higher than 1 in at least 50 samples were retained for downstream analyses. Samples in which the sum of the counts across retained genes was more than 500,000 were kept for downstream analyses.

Normalization was performed using DESeq2 (version 1.36.0 RRID:SCR_015687) following the standard workflow, including normalization by varianceStabilizingTransformation (VST). Due to the use of five distinct sequencing/extraction batches, the resulting batch effect was corrected using ComBat (sva package version 3.44.0 RRID:SCR_012836; see Supplementary Fig. S1A and S1B for pre- and postcorrection principal component analysis; ref. 16).

### Clearseq gene signatures and classifier

A total of eight distinct gene signatures for the molecular subtypes were selected based on previous expression profile studies (Supplementary Table S1) to create a consensus classification system between the ccrcc1–4 system from Beuselinck and colleagues (5) and IMmotion151. The ccrcc3_up and ccrcc3_down signatures were taken from the 35-gene classifier of ccrcc1–4 from Beuselinck and colleagues (17). The cell-cycle signature was adapted from the IMmotion151 subtypes. Ccrcc2_up was composed of genes from ccrcc2_up and ccrcc4_down from the Beuselinck classifier (5), as well as angiogenesis from the IMmotion151 subtypes (10). Both the T-effector signature (IMmotion151 + ccrcc4_up) and ccrcc1&4_up (IMmotion151 fatty acid synthesis, IMmotion151 cell cycle, ccrcc2_down) combine signature information from the original Beuselinck and colleagues (5) classification as well as IMmotion151. Ccrcc1_up combines ccrcc1_up and ccrcc4_down from the original Beuselinck and colleagues (5) with genes from clearcode34_ccB (4).

Signatures were calculated as the median of the expression of the individual genes, and an overview of the signatures informing Clearseq is given as part of Supplementary Table S1. The correlations of the new ccRCC signatures with preexisting signatures are shown in Fig. 1B. The corresponding rules underlying the Clearseq decision-making process are illustrated in Fig. 1C. In short, samples were classified as ccrcc3 in case the following four criteria were true: (i) upper 20th percentile of the ratio of ccrcc3_up (genes upregulated in the original ccrcc3 subtype) to ccrcc3_down (genes downregulated in the original ccrcc3 subtype), (ii) lower 50th percentile of ccrcc2_up, (iii) lower 64th percentile of the cell-cycle signature, and (iv) lower 75th percentile of the T-effector signature. Next, tumors were classified as ccrcc2 in case the ratio of ccrcc2_up to ccrcc1&4_up was higher than 50th percentile (excluding tumors previously classified as ccrcc3). The remaining samples were then assigned to either ccrcc1 or ccrcc4 based on the ratio of the ccrcc4_up to ccrcc1_up, in which samples with the highest 40th percentile of this ratio were classified as ccrcc4 (excluding tumors previously classified as ccrcc3 or ccrcc2; Fig. 1C). The percentiles were defined in order to obtain the same molecular subtype distribution as in the original publication based on fresh-frozen samples.

A Python implementation of the Clearseq1–4 classifier is available via CodeOcean. When applied to larger datasets, cutoff values such as median signature scores may be found in the user’s own data. When applied to smaller datasets or single samples, it is possible to use the cutoffs determined in the provided dataset when preprocessing closely matches the methods used in this article. For improved stability and to enable single-sample classification, a support vector machine (SVM) model trained to identify Clearseq1–4 subtypes, as well as the code used to generate this model, is provided. This model subsets the data to only the genes used in the Clearseq1–4 signatures, rescales these values to have a constant sum per sample to minimize inter-dataset variations, and then calculates gene signature scores before classifying on the basis of these scores. When using any iteration of this classifier, it is advised to match preprocessing methods as closely as possible to the dataset used in this article (i.e., normalization via DESeq2 VST or similar, batch correction via ComBat as needed).

### Gene signatures and immune deconvolution

The IMmotion150 and Javelin Renal 101 signatures were calculated as described in the respective studies (18, 19). Cell type deconvolution was performed using CIBERSORT (20) in absolute mode.

### External validation cohorts

The clinical and transcriptomic (TcgaTargetGtex_rsem_gene_tpm) data of the TCGA-KIRC cohort (https://www.cancer.gov/ccg/research/genome-sequencing/tcga) were downloaded from the Toil RNA-seq Recompute data hub hosted by the UCSC Xena initiative (http://xena.ucsc.edu; refs. 21, 22). The clinical data (S11) and normalized transcriptomic data (S13) from the Javelin Renal 101 cohort were obtained from the published supplementary files (18). The clinical and transcriptomic data (as normalized counts) of the Cancer Research Institute’s iAtlas were downloaded via Synapse (syn10337516; ref. 23). Transcriptomic data were normalized using DESeq2 and batch-corrected via ComBat (16). The dataset was subset for samples from the IMmotion150 cohort. The public single-cell transcriptomics cohort of Bi and colleagues (24) was obtained from the Broad Institute’s single-cell portal under accession SCP1288. Clearseq signatures were calculated as the mean expression of the signature genes per cell. Cell types were aggregated.

### Statistical analysis

Time-to-event analysis was performed with Kaplan–Meier estimates and log-rank tests using the R package “survival” (version 3.3-1 RRID:SCR_021137). For surgical interventions, follow-up started at the time of intervention. Patients were censored at the time of the last disease-free evaluation for DFS and PFS and for the last known time alive for overall survival (OS). All statistical analyses were performed in R (version 4.2.1; RRID:SCR_001905). The Python implementation of the Clearseq classifier and the development of the single-sample SVM classifier were performed using Python (version 3.12.8 RRID:SCR_008394) with the package scikit-learn (version 1.7.2 RRID:SCR_002577).

## Results

### Development of the Clearseq classifier for ccrcc1–4 subtype determination

Within our center, a retrospective cohort of 364 patients with ccRCC was collected, for whom archival FFPE tissues of treatment-naïve primary tumors and/or metastases were available. Bulk transcriptomics was performed on this patient cohort, resulting in a total of 668 primary (*n* = 337) or metastatic (*n* = 331) tumor samples available for expression analysis (Fig. 1A).

We designed a method of classification, further referred to as Clearseq, using eight gene signatures highly characteristic of known RCC subtypes according to previous molecular classifications (see “Materials and Methods”; Supplementary Table S1; refs. 3–5, 10). As such, we aimed to create a consensus classification approach. To further interrogate the relevance of these Clearseq signatures, we performed a correlation analysis within our Leuven cohort between the Clearseq signatures and the current biomarker signatures (2, 18, 19, 25), the signatures from the IMmotion151 subgroups (10), and the signatures from the ClearCode34 classification (4). Ccrcc2_up correlated strongly with angiogenesis and fatty acid oxidation programs. It also showed a substantial association with the ccA signature of ClearCode34. Similarly, ccrcc1&4_up showed a high correlation with cell-cycle and stromal signatures from IMmotion151, as well as ccB from ClearCode34. Meanwhile, the ccrcc4_up signature seemed to capture some lymphocytic pathways (26), highlighted by its coclustering with T-effector signatures and PD-L1 expression. Ccrcc1_up seemed to be related to complement, Ω oxidation, and myeloid programs. These ccrcc1_up patterns, together with findings for ccrcc1&4_up, suggest an overlap in the biological context underlying both ccrcc1 and the IMmotion151 subtypes 5 (Prolif), 6 (Prolif/Stromal), and 3 (complement/Ω oxidation). Finally, signatures related to smaller, less well-defined subtypes, such as the IMmotion151 snoRNA cluster and the ccrcc3 normal-like cluster, were distinct from all the other biomarker signatures. Interestingly, our recently developed tumor low HLA promiscuity (tLHP) signature (25), an ICB-response biomarker, seemed to correlate with both the angiogenesis and T-effector programs (Fig. 1B).

The Clearseq classification system is built on a decision tree that captures consensus trends in all classification systems. It uses dynamic thresholds to assign samples according to the relative proportions of their underlying signatures (see “Materials and Methods” and Fig. 1C). Herein, ccrcc3 samples represent the top 20 percentile of the ccrcc3_up/ccrcc3_down ratio that are also low on angiogenesis (not higher than median ccrcc2_up expression) and not enriched for cell-cycle and T-effector signatures (expression in the lowest 64th and 75th percentiles, respectively). After the removal of ccrcc3, the angiogenesis-enriched subtype ccrcc2 was assigned based on high ccrcc2_up expression relative to ccrcc1&4_up (cell cycle–like programs), as observed in the IMmotion151 study (10). The classifier subsequently identified ccrcc4 samples by a high ccrcc4_up-to-ccrcc1_up ratio. All the remaining samples were assigned to the ccrcc1 group (Fig. 1C).

Additionally, to enable single-sample classification, an SVM-based method was developed, which leverages the Clearseq signatures within each sample and provides confidence values for each proposed classification. This method performs well when recapturing original classifications on a held-out test set (Matthew’s correlation coefficient = 0.9456, *n* = 134, 80/20 training/test split).

### Clinical and transcriptomic characteristics within the Leuven cohort

After allocating each sample to a molecular subtype with the Clearseq classifier, we evaluated the overlap between the subtypes that we previously determined via unsupervised clustering on fresh-frozen tissues described by Beuselinck and colleagues (5, 27). Generally, the Clearseq labels (ccrcc) in FFPE aligned well with the older ccrcc classification in frozen samples for patients who had both FFPE and fresh-frozen tissue samples available (Table 1).

**Table 1.** Overlap (in bold) between ccrcc1–4 subgroups determined by unsupervised transcriptomic clustering on fresh-frozen tissues and those derived by the Clearseq classifier on FFPE-derived transcriptome.

​	*n*	Frozen ccrcc1	Frozen ccrcc2	Frozen ccrcc3	Frozen ccrcc4
Clearseq ccrcc1	24	**15 (63%)**	4 (17%)	0 (0%)	5 (21%)
Clearseq ccrcc2	42	7 (17%)	**33 (79%)**	0 (0%)	2 (5%)
Clearseq ccrcc3	7	2 (29%)	1 (14%)	**2 (29%)**	2 (29%)
Clearseq ccrcc4	18	4 (22%)	5 (28%)	0 (0%)	**9 (50%)**

We subsequently aimed to characterize the clinical and biological features of the different subtypes. A heatmap displaying key gene sets (Fig. 1D) illustrates that ccrcc2 tumors were characterized by enriched angiogenesis, as seen by increased expression of the Javelin101 and IMmotion150 angiogenesis signatures (Fig. 1E; Supplementary Fig. S2A and S2B). Ccrcc4 tumors showed high lymphocyte infiltration, as seen by high expression of both Javelin101 Immuno and IMmotion150 T-effector signatures (Fig. 1E; Supplementary Fig. S2C and S2D). Immune cell type deconvolution (CIBERSORTx) revealed a nonspecific enrichment of CD8^+^ and CD4^+^ T cells, as well as macrophages (M1 and M2; Fig. 1F; Supplementary Fig. S2G and S2H; ref. 28). The latter is also reflected by enrichment in the tLHP and IMmotion150 myeloid signatures (Fig. 1F; Supplementary Fig. S2E and S2F). Morphologically, these tumors had a higher Fuhrman grade and higher sarcomatoid differentiation, reflecting more aggressive tumor behavior (Fig. 1G and H). Ccrcc1 showed enrichment in the IMmotion150 myeloid signature compared with ccrcc2 and ccrcc3, but not compared with ccrcc4 (Fig. 1F; Supplementary Fig. S2F). Compared with ccrcc4, however, ccrcc1 had more infiltration of M0 macrophages and neutrophils (Fig. 1E; Supplementary Fig. S2I). Finally, the ccrcc3 subtype, previously characterized as the “normal kidney–like” subtype, had low expression of both immune- and angiogenesis-related RCC biomarker signatures (Fig. 1F). Immune deconvolution showed a relative increase in mast cells (Fig. 1E; Supplementary Fig. S2J).

For external validation, the Clearseq molecular subtypes were also determined in three external cohorts: TCGA-KIRC, Javelin Renal 101, and IMmotion150 cohorts. Additional analysis within TCGA-KIRC showed an enrichment of the T-cell receptor richness metric in ccrcc4, possibly suggesting higher antigenic polyclonality of T cells within this subgroup (Supplementary Fig. S2K), which is expected due to the prolymphocytic markers (29) in ccrcc4. In Javelin Renal 101, ccrcc4 had the highest proportion of PD-L1–positive samples (93% vs. 68% in ccrcc1, 67% in ccrcc2, and 42% in ccrcc3; *P* < 0.001, Supplementary Fig. S2L).

Next, because the organ tropism of metastases has a well-established prognostic impact (30, 31), we aimed to study the distribution of the subtypes across different metastases in the Leuven cohort. Herein, a clear enrichment of the ccrcc2 subtype was seen in glandular organs, such as the pancreas, adrenal gland, and thyroid [as previously demonstrated by Roussel and colleagues (30) and others (32)], and also in kidney and skin metastases. On the contrary, enrichment of more aggressive subtypes such as ccrcc4 and ccrcc1 was seen in bone, brain, and intestinal metastases, similar to other findings (Fig. 1I; ref. 32).

Finally, we aimed to compare the Clearseq subtypes with the seven subgroups described in the biomarker analyses of the IMmotion151 trial and found that the expression of the respective signatures and their involved genes followed the expected patterns (Supplementary Fig. S3A and S3B).

### Single-cell characteristics of the Clearseq molecular subtypes

Next, we aimed to characterize the cell types driving the broad transcriptional programs captured by the Clearseq molecular subtypes. Utilizing a publicly available single-cell transcriptomic cohort (24), we studied the expression of the Clearseq signatures in each cell type. Herein, we observed that the signatures defining the ccrcc3 subtype (ccrcc3_up and ccrcc3_down) were sufficient to differentiate cancer cells, fibroblasts, and mast cells from the other cell types. For the angiogenesis-enriched ccrcc2 subtype, ccrcc2_up was predominantly expressed by endothelial cells and a subset of cancer cells. The signatures underlying ccrcc4 (ccrcc1&4 up, cell cycle, and T effector) were, expectedly, most expressed by CD8^+^ T cells, myeloid cells, and a subset of cancer cells that showed high proliferation. Finally, the signatures defining ccrcc1 were most expressed by cancer cells (ccrcc1_up) and myeloid cells (ccrcc1.4_up; Fig. 1J; Supplementary Fig. S4A–S4H). In conclusion, each ccrcc class captured cancer cell–intrinsic properties and also simultaneously captured different components of the TME, thereby emphasizing its broad applicability.

### Molecular subtypes show prognostic impact in surgical settings

**Figure 2.**
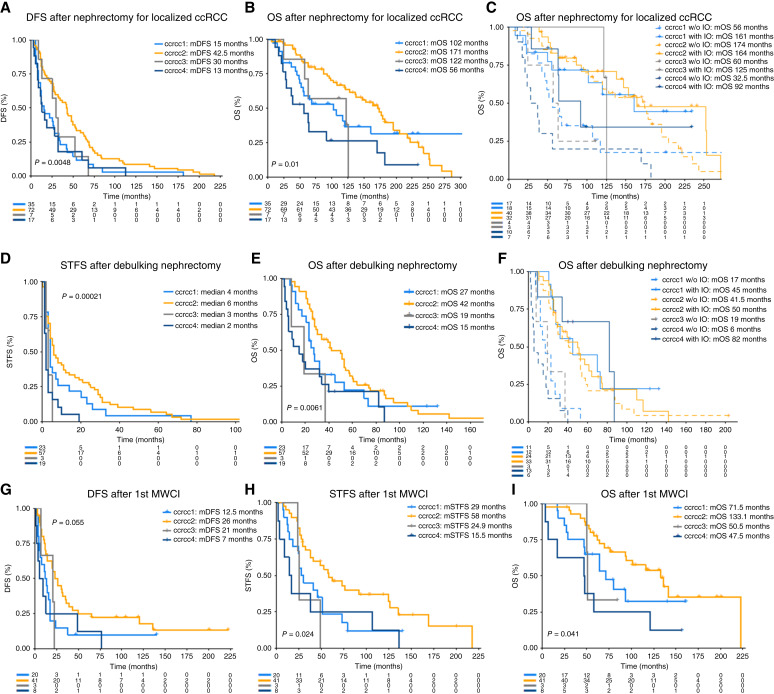
Clearseq molecular subtypes are prognostic across different surgical contexts. **A** and **B,** DFS (**A**) and OS (**B**) after nephrectomy for localized ccRCC by molecular subtype. **C,** OS after nephrectomy for localized ccRCC by molecular subtype and by access to ICB before death. **D** and **E,** STFS (**D**) and OS (**E**) after debulking nephrectomy by molecular subtype. **F,** OS after debulking nephrectomy by molecular subtype and by access to ICB before death. **G** and **H,** DFS (**G**) and STFS (**H**) after MWCI by molecular subtype. **I,** OS after MWCI by molecular subtype.

In the Leuven cohort, 132 patients with localized ccRCC underwent nephrectomy with curative intent (Supplementary Table S2). DFS was significantly (*P* = 0.0045) different between molecular subgroups, with the longest DFS for patients with ccrcc2 tumors [median DFS (mDFS) of 30 months], as opposed to patients with ccrcc4 tumors, which relapsed early (mDFS 13 months; Fig. 2A). Similarly, this impact was also seen on OS after nephrectomy (Fig. 2B). The Clearseq classification remained significant for DFS and OS in a multivariable model, including T, N, and Fuhrman grade (Supplementary Table S3). Clearseq also captured interesting features relevant for the treatment of metastatic disease. When ICB-containing regimens as treatment for metastatic disease were evaluated separately from those that did not, notable differences in outcomes could be seen. Our data suggest that the more aggressive molecular subtypes (ccrcc1 and ccrcc4) benefit more from ICBs than those with an angiogenesis-driven subtype (ccrcc2; Fig. 2C).

A total of 104 patients were included who underwent a debulking nephrectomy for metastatic disease. Each of these patients later received systemic therapy (Supplementary Table S4). Consistent with the more aggressive nature of these tumors, the median systemic therapy–free survival (mSTFS) of patients with ccrcc4 tumors was only 2 months after debulking nephrectomy, whereas mSTFS was 6 months in patients with ccrcc2 tumors (Fig. 2D). Similar to our observations after nephrectomy in localized disease, the molecular subtypes also correlate with OS after debulking nephrectomy, and again, this effect was mainly present in patients who did not receive ICB before death (Fig. 2E and F). The Clearseq classification remained significant for STFS in a multivariable analysis with T, N, and Fuhrman grade; however, it was not significant for OS (Supplementary Table S3).

The final surgical cohort is a series of 73 patients who underwent a MWCI (Supplementary Table S5). When classified by the molecular subtype as determined on their primary tumor, patients with ccrcc2 have the longest DFS, STFS, and OS (Fig. 2G–I). Notably, we again observe that the impact on OS becomes insignificant when these patients are later treated with ICB. In a multivariable model with the Leuven-Udine categories (33), DFS and OS, but not STFS, remained significantly correlated with the Clearseq classification (Supplementary Table S6). Overall, the strong prognostic impact observed in previous studies (6, 7) was confirmed using the Clearseq classification.

### Predictive value of molecular classification for VEGFR-TKI treatment

**Figure 3.**
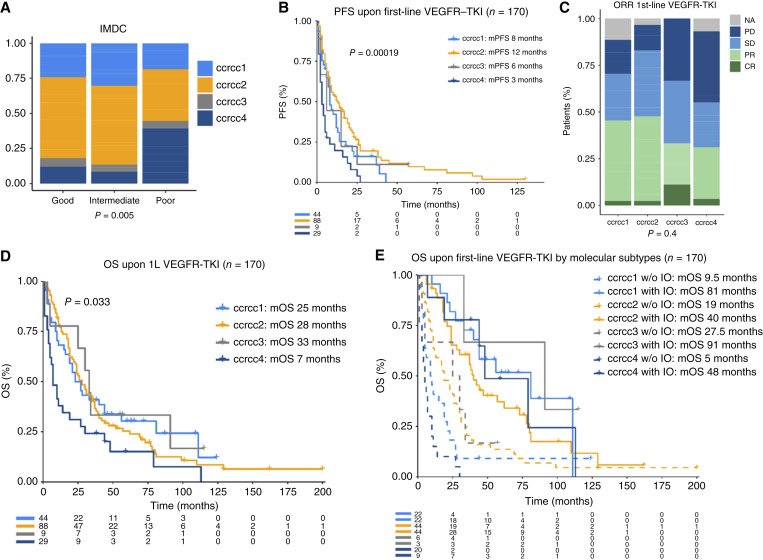
Clearseq molecular subtypes have predictive value for angiogenesis inhibitors. **A,** IMDC risk groups in patients treated with VEGFR-TKI in the first line by molecular subtype. **B,** Kaplan–Meier curves showing PFS upon first-line treatment with VEGFR-TKI by molecular subtype. **C,** Bar chart of best response upon first-line treatment with VEGFR-TKIs by molecular subtype. **D,** Kaplan–Meier curves showing OS upon first-line treatment with VEGFR-TKIs by molecular subtype. **E,** Kaplan–Meier curves showing OS upon first-line treatment with VEGFR-TKIs by molecular subtype and whether patients received ICB before death.

Next, we aimed to evaluate whether the Clearseq classification also showed the predictive impact for VEGFR-TKIs seen in previous studies (5). A total of 170 patients were available who received VEGFR-TKI as first-line treatment. Of these, 90 patients were treated with sunitinib, 71 with pazopanib, and 9 with sorafenib (Supplementary Table S7). Consistent with our previous results in fresh-frozen tissues (27, 34), patients with the ccrcc4 subtype were enriched in International Metastatic Renal Cell Carcinoma Database (IMDC) poor risk (Fig. 3A). The angiogenesis-enriched ccrcc2 group correlated with improved PFS, whereas the inflamed ccrcc4 subtype had the shortest PFS (Fig. 3B). RR was numerically higher in ccrcc1 and ccrcc2 tumors compared with ccrcc4 tumors (44% and 47% vs. 31%, *P* = 0.5, Fig. 3C). Consistent with our results in the surgical settings, molecular subtypes demonstrated an impact on OS in the subgroup of patients who were unable to access ICB in later lines, whereas outcomes became comparable in patients who did (Fig. 3D and E). These results indicate that the ICBs primarily improve the prognosis in patients with ccrcc1 and ccrcc4 tumors, with the most important benefit of ICB in ccrcc4 tumors. These results remained stable when reanalyzed with multiparametric approaches accounting for IMDC risk groups (Supplementary Table S8).

Another important clinical question was the effectiveness of VEGFR-TKIs after previous systemic treatment. In our cohort, 61 patients were included who were treated with VEGFR-TKIs in two consecutive treatment lines (Supplementary Table S9). RRs were low overall, but patients with the angiogenic ccrcc2 subtype had the numerically highest ORR and PFS (ORR 12%; mPFS 6 months) when compared with other subtypes (Supplementary Fig. S5A and S5B). Patients with the ccrcc4 subtype, comparatively, had worse OS (Supplementary Fig. S5C), which remained significant in multivariable analysis with IMDC risk groups (Supplementary Table S8). Finally, we evaluated the impact of the Clearseq classification on patients treated with VEGFR-TKIs in the second-line after treatment with ICB in the first line (ipilimumab/nivolumab in 19, avelumab in 1, axitinib/pembrolizumab in 1, and nivolumab in 1 patient), which reflected the current treatment landscape of ccRCC (Supplementary Table S10). Only 22 patients were available for these exploratory analyses; nevertheless, we observed a numerically longer OS and highest ORR in ccrcc2 tumors (Supplementary Fig. S5D–S5F). Given the insufficient number of events, multivariable analysis was not performed.

### ICB overcomes the negative prognosis of ccrcc4 tumors

**Figure 4.**
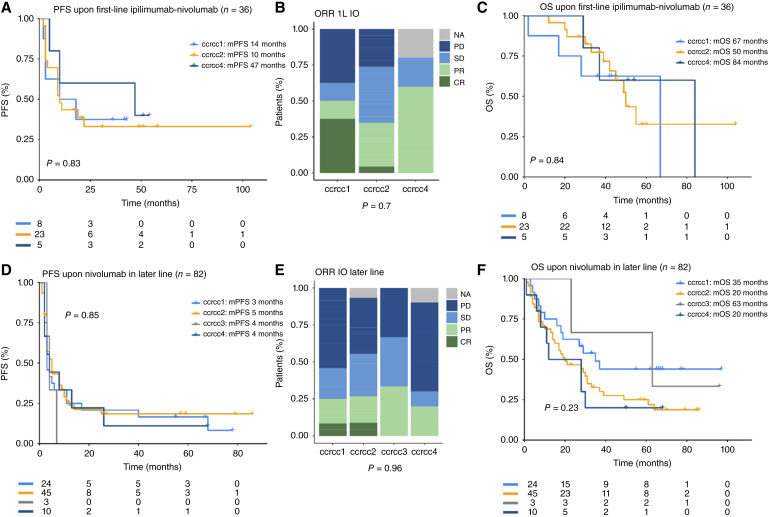
Clearseq molecular subtypes have predictive value for immune checkpoint inhibitors. **A,** Kaplan–Meier curves showing PFS upon first-line therapy with ipilimumab–nivolumab by molecular subtype. **B,** Bar chart of best response upon first-line treatment with ipilimumab–nivolumab by molecular subtype. **C,** Kaplan–Meier curves showing OS upon first-line treatment with ipilimumab–nivolumab by molecular subtype. **D,** Kaplan–Meier curves showing PFS upon treatment with nivolumab by molecular subtype. **E,** Bar chart of best response upon treatment with nivolumab by molecular subtype. **F,** Kaplan–Meier curves showing OS upon treatment with nivolumab by molecular subtype.

Finally, we aimed to study the impact of the subtypes for ICB treatment, as one of the cornerstones of current-day ccRCC treatment. A total of 36 patients treated with ipilimumab–nivolumab as first-line treatment were available for transcriptomic analysis (patient characteristics in Supplementary Table S11). Within this smaller cohort, no ccrcc3 tumors were identified. In this exploratory analysis, we observed a striking difference compared with the outcome upon first-line TKI treatment. No statistically significant difference between the groups was identified. Ccrcc4 had the numerically longest PFS (Fig. 4A), the highest RR (ORR 60% vs. 50% in ccrcc1 and 39% in ccrcc2, Fig. 4B), and the longest OS (Fig. 4C). Within a cohort of 82 patients treated with nivolumab in a later line (patient characteristics in Supplementary Table S12), comparable results were observed. Patients with adverse prognostic subtypes had similar PFS, ORR, and OS as those with favorable prognostic subtypes. This finding remains valid upon bivariate analysis with IMDC prognostic groups (Fig. 4D–F; Supplementary Table S13).

### External validation for surgical (TCGA-KIRC) and systemic (Javelin Renal 101; IMmotion150) therapies

**Figure 5.**
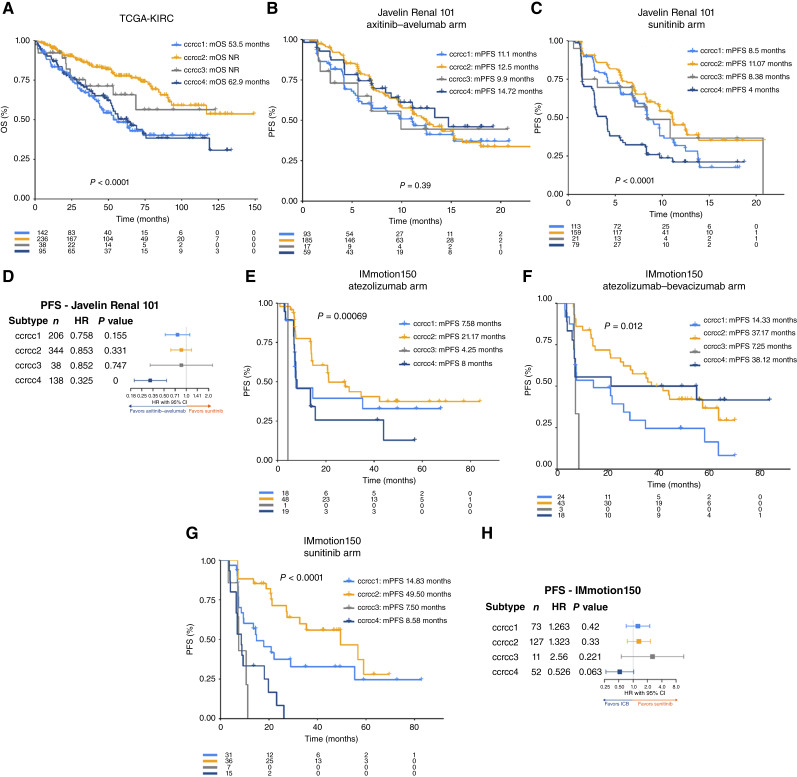
Validation of molecular subtypes in external cohorts. **A,** Kaplan–Meier curves showing OS after nephrectomy in the TCGA-KIRC cohort. **B** and **C,** Kaplan–Meier curves showing PFS in the avelumab–axitinib (**B**) arm and the sunitinib (**C**) arm of the Javelin Renal 101 cohort. **D,** Forest plot showing HR and 95% CI of univariate (UVA) Cox proportional hazards models comparing avelumab–axitinib vs. sunitinib-treated arms of the Javelin Renal 101 cohort. **E–G,** Kaplan–Meier curves showing PFS in the atezolizumab (**E**), atezolizumab–bevacizumab (**F**), and sunitinib (**G**) arms of the IMmotion150 cohort. **H,** Forest plot showing HR and 95% CI of UVA Cox proportional hazards models comparing ICB arms (atezolizumab + atezolizumab–bevacizumab) vs. sunitinib-treated arm of the IMmotion150 cohort.

To provide external validation of our findings, we determined the Clearseq subtypes in three external cohorts. In the TCGA-KIRC cohort, ccrcc2 patients had improved OS after nephrectomy, whereas ccrcc1 and ccrcc4 subtypes performed the worst (Fig. 5A). In the Javelin Renal 101 phase III trial cohort, comparing avelumab–axitinib (anti–PD-L1/VEGFR-TKI) with sunitinib (VEGFR-TKI) as first-line treatment, there were again significant differences. In the avelumab–axitinib arm, PFS is comparable across subgroups (Fig. 5B), as opposed to a markedly worse outcome for the ccrcc4 subtype in the sunitinib arm (*P* < 0.0001; Fig. 5C). Across subgroups, the HR for PFS favored the avelumab–axitinib arm over sunitinib, but the magnitude of benefit was most substantial in the ccrcc4 subgroup (HR, 0.325; *P* < 0.001; Fig. 5D). In the IMmotion150 phase II trial cohort, the subtypes significantly stratified patients across the three treatment arms (atezolizumab, Fig. 5E; atezolizumab–bevacizumab, Fig. 5F; sunitinib, Fig. 5G). Remarkably, when comparing PFS in the ICB-treated arms with the sunitinib arm, ccrcc1, ccrcc2, and ccrcc3 numerically favor sunitinib (HR >1), whereas ccrcc4 seems to derive more benefit from ICB-containing regimens, albeit nonsignificantly (Fig. 5H).

### Interplay of antigenicity with molecular subtypes

**Figure 6.**
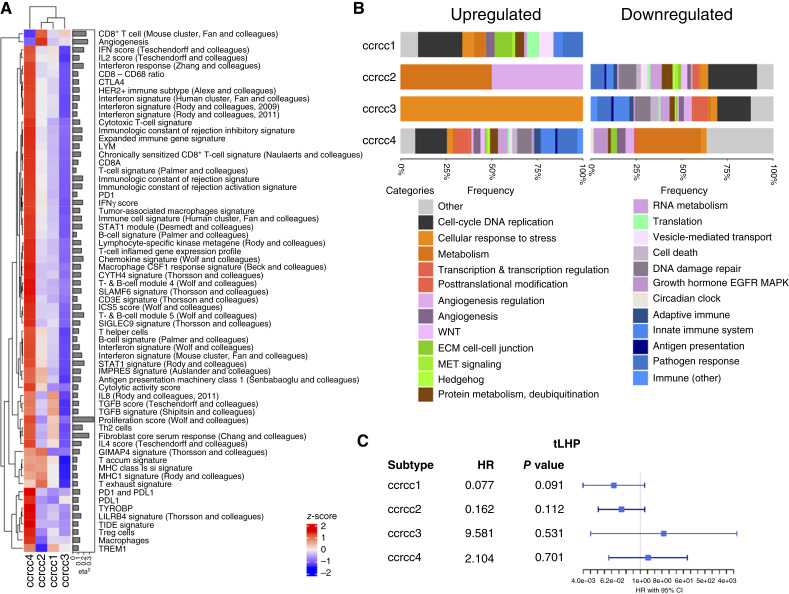
Immune tumor microenvironment of Clearseq subtype. **A,** Heatmap displaying the expression of immune-oncology gene signatures (selected for η^2^ >0.06). Columns and rows are clustered (complete method). **B,** Bar chart displaying enriched GSEA REACTOME pathways at FDR <0.25 (<0.42 for ccrcc1). No signatures were downregulated in ccrcc1 at FDR <0.42. **C,** Forest plot showing HR and 95% CI of UVA Cox proportional hazards models of the correlation of the tLHP signature with OS in the Leuven cohort.

Although current transcriptomic subgroup-based classifications can identify an inflamed subgroup with poor prognosis at baseline and apparent benefit from ICB-containing regimens, the heterogeneity in patient response to ICB treatment remains elusive (35). To further delineate the immune TME (iTME) in relation to the consensus ccrcc subtypes, we studied the distribution of published immune-oncology gene signatures (Fig. 6A; refs. 25, 36). When selecting for signatures differently expressed at a moderate effect size (i.e., η^2^ >0.06), we observed a nonspecific enrichment of nearly all of these signatures in the ccrcc4 subtype. This suggests that the iTME of ccrcc4 is characterized by nondirectional inflammation rather than an organized immune response (29, 37). Additionally, ccrcc1 is relatively enriched in wound healing signatures, whereas ccrcc2 showed tendencies toward angiogenesis and antigenicity-related signatures consistent with its definition (Fig. 6A). To further characterize pathways enriched in these subtypes, we performed gene set enrichment analysis leveraging the REACTOME database (Fig. 6B). Pathways enriched at FDR <0.25 (<0.42 for ccrcc1) were selected and divided into broad categories. For both ccrcc1 and ccrcc4, cell cycle–related pathways take up the largest proportion, followed by pathways related to the response to pathogens. In ccrcc4, an important proportion was taken by the innate immune system as well as the adaptive immune system, whereas the latter was hardly present in any other subtype. Next, we questioned how the recently developed tLHP signature behaves within each subtype. We observed a trend toward improved OS in ccrcc1 (*n* = 31) and ccrcc2 (*n* = 68) tumors with higher tLHP expression, whereas in ccrcc4 (albeit with smaller numbers, *n* = 14), this is not the case (Fig. 6C).

## Discussion

Molecular classifications based on unsupervised clustering of tumor transcriptomic data have strongly advanced insights into ccRCC tumor biology over the past 15 years. The consistently identified archetypes of an angiogenesis-enriched subgroup, a subgroup with strong T cell infiltration and a subgroup with enhanced cell cycle and myeloid infiltration have demonstrated prognostic value, as well as predictive value for current systemic treatments. Nevertheless, the determination of these subtypes in novel cohorts remains challenging, and their value for current first-line treatments (in particular, ipilimumab–nivolumab) has not yet been demonstrated. With this study, we aimed to develop an easily replicable classifier for the ccrcc1–4 classification, which reflects the insights gained by various others over the past 15 years. Moreover, we aimed to study it across different ccRCC treatment settings in our real-world cohorts, as well as in external validation cohorts, and provide additional insights through signature analysis at the single-cell level.

We developed an easy-to-reproduce gene expression–based classifier of ccRCC tumors, called Clearseq1–4, capable of detecting the ccrcc1–4 molecular subtypes, including their previously described clinical and molecular features, and based on the expression of a reduced number of genes. Moreover, using a public single-cell cohort, we provided further insights about these subtypes at the single-cell level, in particular which cell types might be important drivers of subtype-determining features. We subsequently demonstrated the impact of the Clearseq classification on clinical outcomes. In post-nephrectomy as well as MWCI settings, we comprehensively showed the strong prognostic impact of the Clearseq classifier. The post-nephrectomy impact was externally validated in the TCGA-KIRC cohort. In the context of systemic therapy with VEGFR-TKI, the proangiogenic ccrcc2 tumors show improvement in PFS and ORR. The effect on OS, however, was more distinct in patients not receiving ICB in later lines but became nonsignificant when patients did have access, indicating that ICB overcame the aggressive behavior of the TKI-nonresponding tumors. Finally, analysis of ICB-treated cohorts showed that patients with the ccrcc4 subtype derived comparatively more treatment benefit and now had comparable outcomes with other subtypes. To provide cross-cohort validation of the classifier, we also confirmed its impact in two external cohorts treated with anti–PD-L1/antiangiogenic combination therapies (IMmotion150 and Javelin Renal 101 cohorts). Within these cohorts, ccrcc4 again benefited comparatively more from the addition of an ICB to an angiogenesis inhibitor. The classifier can be applied to relatively small tumor series.

These results are consistent with the results of the previous version of the ccrcc1–4 classification, as developed through unsupervised clustering of transcriptomic data from fresh-frozen tissues. We showed correlation with DFS and OS after nephrectomy for localized ccRCC (6), as well as after complete metastasectomy (7). For systemic treatments, the angiogenesis-enriched subtype performed better on sunitinib (5), as well as pazopanib (27). In a biomarker analysis of the CheckMate009 study, the ccrcc1–4 subgroups were also determined from baseline tumor samples of 56 patients with advanced RCC treated with nivolumab as a single agent. Within this clinical trial cohort, the molecular characteristics were confirmed, that is, with ccrcc2 tumors displaying high angiogenesis and ccrcc4 tumors having high T cell/inflammation–related scores. Patients with ccrcc4 tumors had the highest RR (47%, *P* = 0.013 relative to non-ccrcc4–like). Angiogenesis and the ccrcc2 subtype showed a negative association with nivolumab response. The authors did not report data on PFS or OS (38). The distinct TME characteristics of the ccrcc1–4 classification (5, 27) inspired the BIONIKK trial, which is a phase II biomarker-driven trial designed on the hypothesis of differential responses to ICB and VEGFR-TKIs between subtypes: Patients with ccrcc1 or ccrcc4 were randomized between ipilimumab/nivolumab and nivolumab alone, whereas the angiogenesis-enriched ccrcc2 and normal-like ccrcc3 were randomized between ipilimumab/nivolumab and VEGFR-TKI. Herein, the authors observed that the proangiogenic subtype ccrcc2 had a high ORR with both treatments but had a longer mPFS on VEGFR-TKI compared with ipilimumab-nivolumab. In the ccrcc4 subtypes, comparably high RR and PFS were observed with both nivolumab and ipilimumab-nivolumab, whereas the ccrcc1 subtype seemed to benefit from the addition of ipilimumab to nivolumab (13).

As previously discussed, other classifications have been developed as well. For instance, four subgroups were identified in the TCGA-KIRC cohort (8). The m1 cluster, which resembled ccrcc2 with upregulation of metabolism pathways and enrichment of *PBRM1* mutations, was correlated with longer survival after nephrectomy. In contrast, m3 and m4 showed upregulations of MYC targets and the cell cycle, with m4 also having upregulation of IFN pathways. These subtypes, corresponding to ccrcc1 and ccrcc4, had worse survival (8). Within the COMPARZ cohort of patients treated with VEGFR-TKIs, four subtypes were discovered, which also had high similarity to the ccrcc1–4 classification and confirmed the superior response to VEGFR-TKIs within the angiogenesis-enriched subgroup. Most recently, seven transcriptomic subsets were identified in the IMmotion151 biomarker analyses. These showed differential responses to atezolizumab/bevacizumab versus sunitinib. More specifically, an increased benefit from the addition of ICB was observed within the T-effector/proliferative subtype, which corresponds to ccrcc4. These subsets were externally validated in cohorts treated with avelumab–axitinib (Javelin Renal 101; ref. 11), atezolizumab–bevacizumab (IMmotion150; ref. 10), and nivolumab (CheckMate025; ref. 12). Additionally, the prospective phase II OPTIC was designed to prospectively determine these subtypes in metastatic patients with ccRCC and to randomize them between dual ICB versus ICB/VEGFR-TKI as first-line treatment depending on the IMmotion151 molecular subtype (NCT05361720; ref. 14). Ultimately, the findings of these other classifications consistently align with our own. This underlines the strength of the biomarker potential of the Clearseq1–4 classification, particularly in surgical settings and VEGFR-TKI-treated patients.

Very recently, in the pivotal phase III study comparing lenvatinib/pembrolizumab with sunitinib, the impact of six molecular subtypes, close to the IMmotion151 subtypes, on outcomes in both treatment arms was studied. Interestingly, the authors developed a classifier in a manner similar to Clearseq, using several gene expression panels (angiogenesis, stroma/EMT/TGFbeta, T-cell inflamed gene expression profile, and proliferation) and specific cutoffs (upper tertile, median, 75%) in order to classify the samples in four steps into the six subgroups (39).

Nevertheless, with the current evidence available, the molecular subtypes do not seem to provide a comprehensive explanation for the heterogeneity observed in ICB response in patients with ccRCC. This is likely explained by the fact that these classifications are based on large-scale, broad transcriptomic patterns, which are mostly driven by cancer cells, stromal cells, and the resident immune infiltration patterns. The lack of specificity may fail to adequately capture the qualitative immunologic nuances, which are key to distinguishing ICB responders from nonresponders, as well as not capturing the dynamic aspects of an antitumoral immune response, which take place in lymph nodes and in circulation. This is reflected by the distribution of common immune-oncology signatures, which point toward a nondirectional inflammation in ccrcc4, whereas current RCC ICB biomarker signatures, such as tLHP, were enriched in both ccrcc2 and ccrcc4. As such, the inclusion of additional evidence from integrated omic studies will likely further enhance patient stratification across subgroups. For angiogenic-directed therapies, increased vascularization is a known biomarker for response (9, 34), which seems much more reliably captured by molecular subtypes, as well as being more consistent, which results in a stronger biomarker value for angiogenesis inhibitors. Additionally, patients with a more angiogenically enriched subtype have a better prognosis, which is further improved by VEGFR-TKIs, leading to marked differences with aggressive subtypes that are nonresponding. Nevertheless, this remains a limitation for the use of molecular subtypes for current first-line therapies as it cannot exclude patients from one therapy or another.

Other limitations include potential heterogeneity between metastases and their primary tumor, as we used the subtype of the primary tumor for stratification (15). Intratumor heterogeneity is another possible confounder. Due to small numbers of patients with available transcriptomic data, we were not able to include a first-line ICB/VEGFR-TKI cohort from our institution. Globally, although the total series includes more than 300 patients, the subgroups used for the different outcome analyses in distinct clinical settings, from early stage to metastatic disease, and with a mix of systemic therapies in the metastatic setting, are relatively small. Moreover, after the ccrcc1–4 classification was developed, and based on this classification, the seven IMmotion151 transcriptomic clusters were described. Direct comparison between the current classification and the IMmotion151 subtypes has not yet been performed. Nevertheless, we aim to perform this in a future project, upon gaining access to the annotated IMmotion151 transcriptomic data and classifier. Although Clearseq1–4 can predict the efficacy of ICBs to some extent, we need to add additional markers to improve the accuracy of the marker’s predictive value for response to ICBs. Work is currently ongoing. Finally, validation in a larger cohort would be needed to establish this method of categorizing or making therapy decisions in ccRCC.

In comparison with existing methods of classifying ccRCC expression profiles, Clearseq1–4 has evident clinical advantages. The ccA-ccB classification, as well as the IMmotion151 clusters (or the IMmotion150 classification) used by the OPTIC trial and the ccrcc1–4 classification, used by the BIONIKK trial, are comparable ways to classify clear-cell RCCs. However, the ccA-ccB classification only divides the tumors into two categories, which is a low number of categories, allowing only classification between more indolent and more aggressive tumors. The IMmotion151 classification provides a more granular division into seven clusters compared with Clearseq1–4. However, the snord cluster (cluster 7) is very small and unreliably replicated in external datasets, and the distinct molecular characteristics of clusters 3, 5, and 6 do not seem to translate into important differences in terms of predictive or prognostic impact when compared among each other. The strength of our classification into four subgroups is that the resulting groups show clear outcome differences in all clinically relevant settings: after nephrectomy, after debulking nephrectomy, after metastasectomy, and upon VEGFR-TKIs and ICB in the metastatic setting. Furthermore, the classifier can be applied to sequencing data from FFPE samples, which can be performed in routine clinical pathology laboratories, even at limited sample numbers, as long as preprocessing is similar to that used in this study.

### Conclusion

We developed an easy-to-reproduce ccRCC classification system, called Clearseq1–4, compatible with FFPE-fixed tissues, which builds on conserved information across all currently published classification systems. The Clearseq classifier demonstrated prognostic value, as well as predictive value for current systemic treatments, mainly for VEGFR-TKIs but, to a lesser extent, also for ICBs.

## Supplementary Material

Suppl. Figure 1 Principal component analysis

Suppl. Figure 2 Pathological and molecular characteristics of Clearseq molecular subtypes.

Suppl. Figure 3 Distribution of IMmotion151 signatures across Clearseq subtypes.

Suppl. Figure 4 Single-cell characterization of Clearseq signatures.

Suppl. Figure 5 Response to angiogenesis inhibitors in later line by Clearseq molecular subtypes

Suppl. Table 1 Clearseq signatures (A) and comparison with genes included in our previous model (B) based on fresh frozen samples

Suppl. Table 2 Characteristics of patients who received nephrectomy for localised ccRCC

Suppl. Table 3 Multivariable models for nephrectomy for localised ccRCC and debulking nephrectomy

Suppl. Table 4 Characteristics of patients who received debulking

Suppl. Table 5 Characteristics of patients who received metastasectomy with curative intent

Suppl. Table 6 Multivariable models within metastasectomy with curative intent cohort

Suppl. Table 7 Patient characteristics of the VEGFR-TKI in first-line cohort

Suppl. Table 8 Multivariable cox proportional hazards models

Suppl. Table 9 Patient characteristics in the sequential TKI cohort

Suppl. Table 10 Patient characteristics for the VEGFR-TKI after ICB cohort

Suppl. Table 11 Patient characteristics of the dual ICB cohort

Suppl. Table 12 Patient characteristics of the cohort treated with nivolumab in later line

Suppl. Table 13 Bivariable cox proportional hazards models

## Data Availability

All our own data generated and analyzed during this study are publicly available at codeOcean (https://codeocean.com/capsule/4435314/tree/v1). We also assessed publicly available databases. Clinical, genomic, and normalized transcriptomics data from the JAVELIN 101 cohort were obtained from the supplementary files of the original publication (18). Survival and transcriptomic data for the TCGA-KIRC cohort were obtained from Xena (http://xena.ucsc.edu). Clinical and transcriptomic data of IMmotion 150 were obtained via the Cancer Research Institute’s iAtlas Synapse (syn10337516).
