# Supplementary material for: Prognostic and Predictive Value of the Clearseq1–4 Tumor Microenvironment Classification in Localized and Metastatic Clear-Cell Renal Cell Carcinoma
Source: Cancer Res Commun. 2026 Apr 20;6(4):884–97. doi: 10.1158/2767-9764.CRC-25-0548 (PMC13095203; doi:10.1158/2767-9764.CRC-25-0548)
Supplement: Suppl. Figure 1 — Principal component analysis [file crc-25-0548_suppl.figure_1_suppsf1.docx]

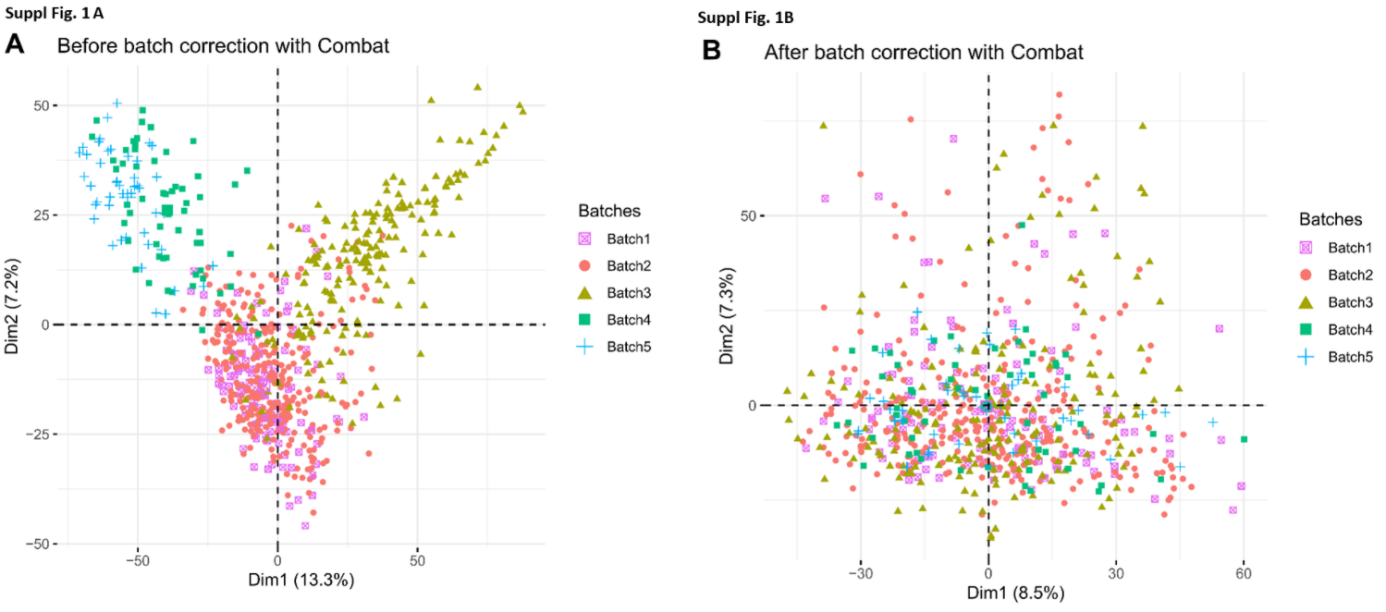


**Suppl. Fig. 1 Principal component analysis (PCA) before and after batch correction. A,** PCA plot of normalized RNA-seq data before correction of batch effect. **B,** PCA plot of normalized RNA-seq data after correction of batch effect using ComBat (sva package v.3.44.0 RRID:SCR_012836).
