# Supplementary material for: Prognostic and Predictive Value of the Clearseq1–4 Tumor Microenvironment Classification in Localized and Metastatic Clear-Cell Renal Cell Carcinoma
Source: Cancer Res Commun. 2026 Apr 20;6(4):884–97. doi: 10.1158/2767-9764.CRC-25-0548 (PMC13095203; doi:10.1158/2767-9764.CRC-25-0548)
Supplement: Suppl. Figure 2 — Pathological and molecular characteristics of Clearseq molecular subtypes. [file crc-25-0548_suppl.figure_2_suppsf2.docx]

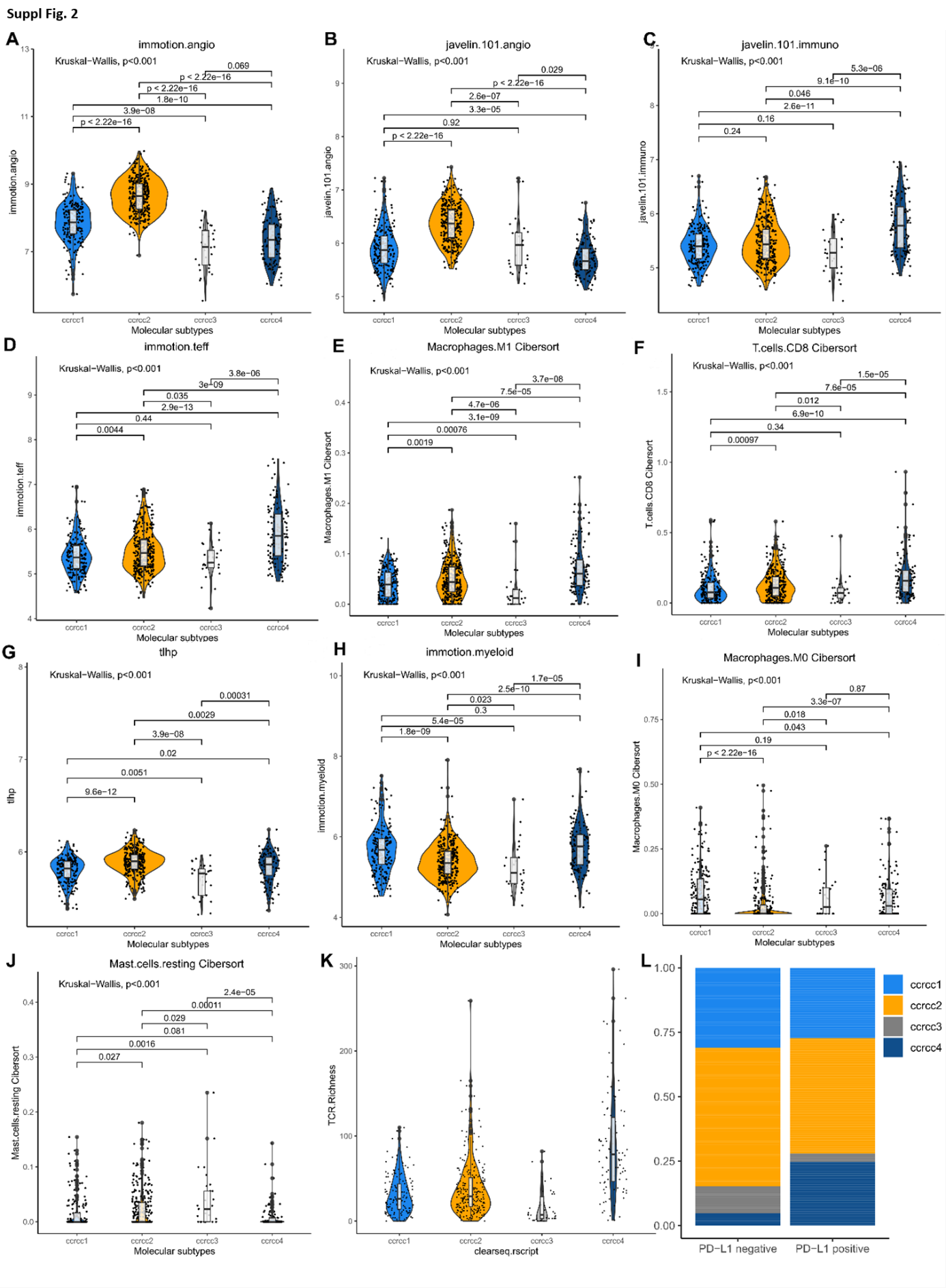


**Suppl. Fig. S2 Pathological and molecular characteristics of Clearseq molecular subtypes.** **A-D,** Violin- and boxplots showing expression of IMmotion150 angio (A), Javelin101 angio (B), Javelin101 Immuno (C), IMmotion150 T effector (D) by Clearseq molecular subtypes. **E-F,** Violin- and boxplots showing CIBERSORTx deconvoluted cell types: M1 macrophages (E) and CD8^+^ T cells (F) by Clearseq molecular subtypes. **G-H,** Violin- and boxplots showing tLHP (G) and IMmotion myeloid score (H) by Clearseq molecular subtypes. **I-J:** Violin- and boxplots showing CIBERSORTx deconvoluted cell types: M0 macrophages (I), and resting mast cells (J) by Clearseq molecular subtypes. **K,** Violin- and boxplots showing TCR richness in TCGA-KIRC by molecular subtypes. **L,** Bar charts showing PD-L1 IHC staining in Javelin Renal 101 by molecular subtypes.
