# Supplementary material for: Prognostic and Predictive Value of the Clearseq1–4 Tumor Microenvironment Classification in Localized and Metastatic Clear-Cell Renal Cell Carcinoma
Source: Cancer Res Commun. 2026 Apr 20;6(4):884–97. doi: 10.1158/2767-9764.CRC-25-0548 (PMC13095203; doi:10.1158/2767-9764.CRC-25-0548)
Supplement: Suppl. Figure 3 — Distribution of IMmotion151 signatures across Clearseq subtypes. [file crc-25-0548_suppl.figure_3_suppsf3.docx]

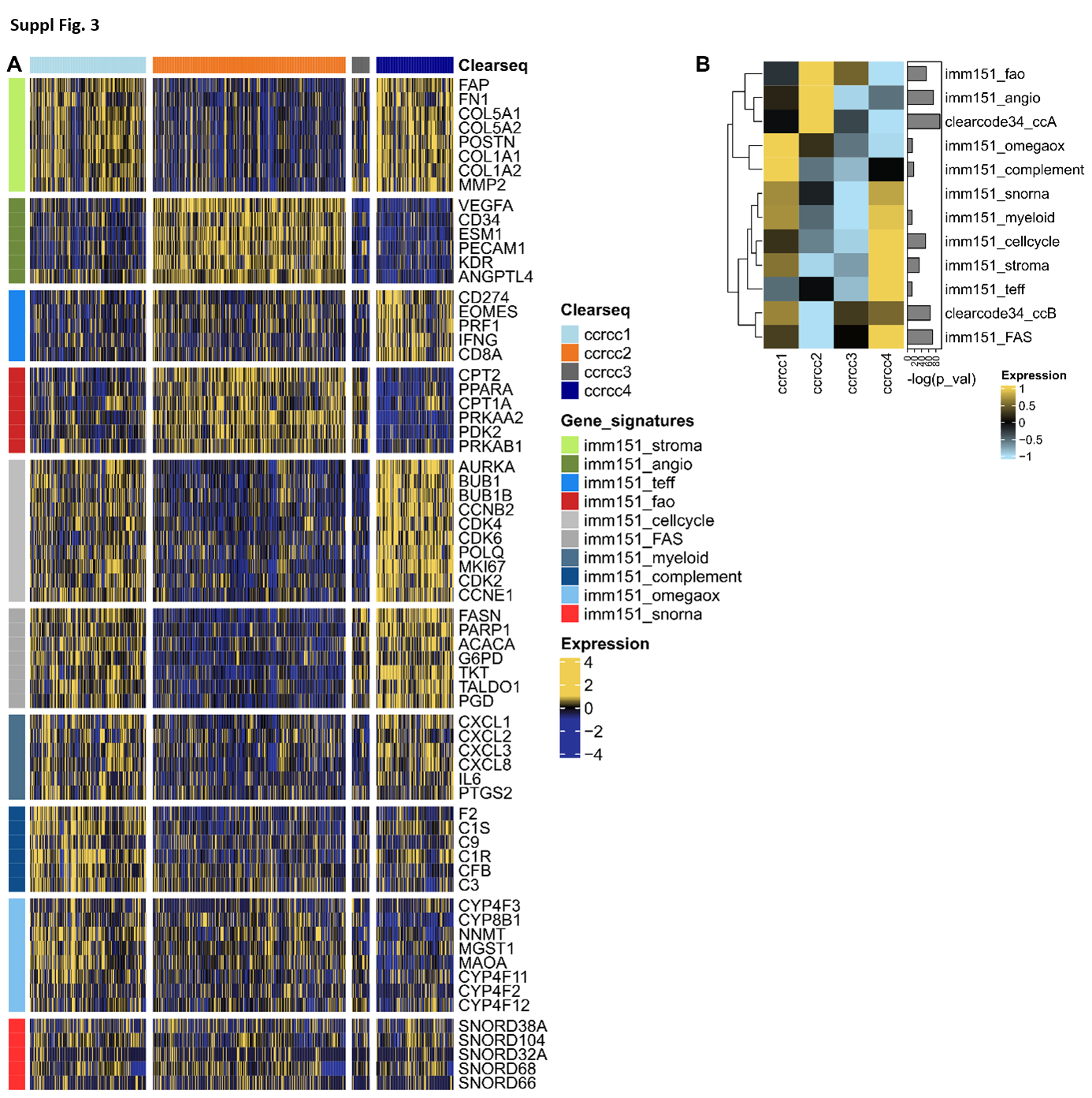


**Suppl. Fig. 3 Distribution of IMmotion151 signatures across Clearseq subtypes.** A, Heatmap showing expression of genes included in IMmotion151 signatures. B, Heatmap showing expression of IMmotion151 and ClearCode34 signatures.
