# Supplementary material for: Prognostic and Predictive Value of the Clearseq1–4 Tumor Microenvironment Classification in Localized and Metastatic Clear-Cell Renal Cell Carcinoma
Source: Cancer Res Commun. 2026 Apr 20;6(4):884–97. doi: 10.1158/2767-9764.CRC-25-0548 (PMC13095203; doi:10.1158/2767-9764.CRC-25-0548)
Supplement: Suppl. Figure 4 — Single-cell characterization of Clearseq signatures. [file crc-25-0548_suppl.figure_4_suppsf4.docx]

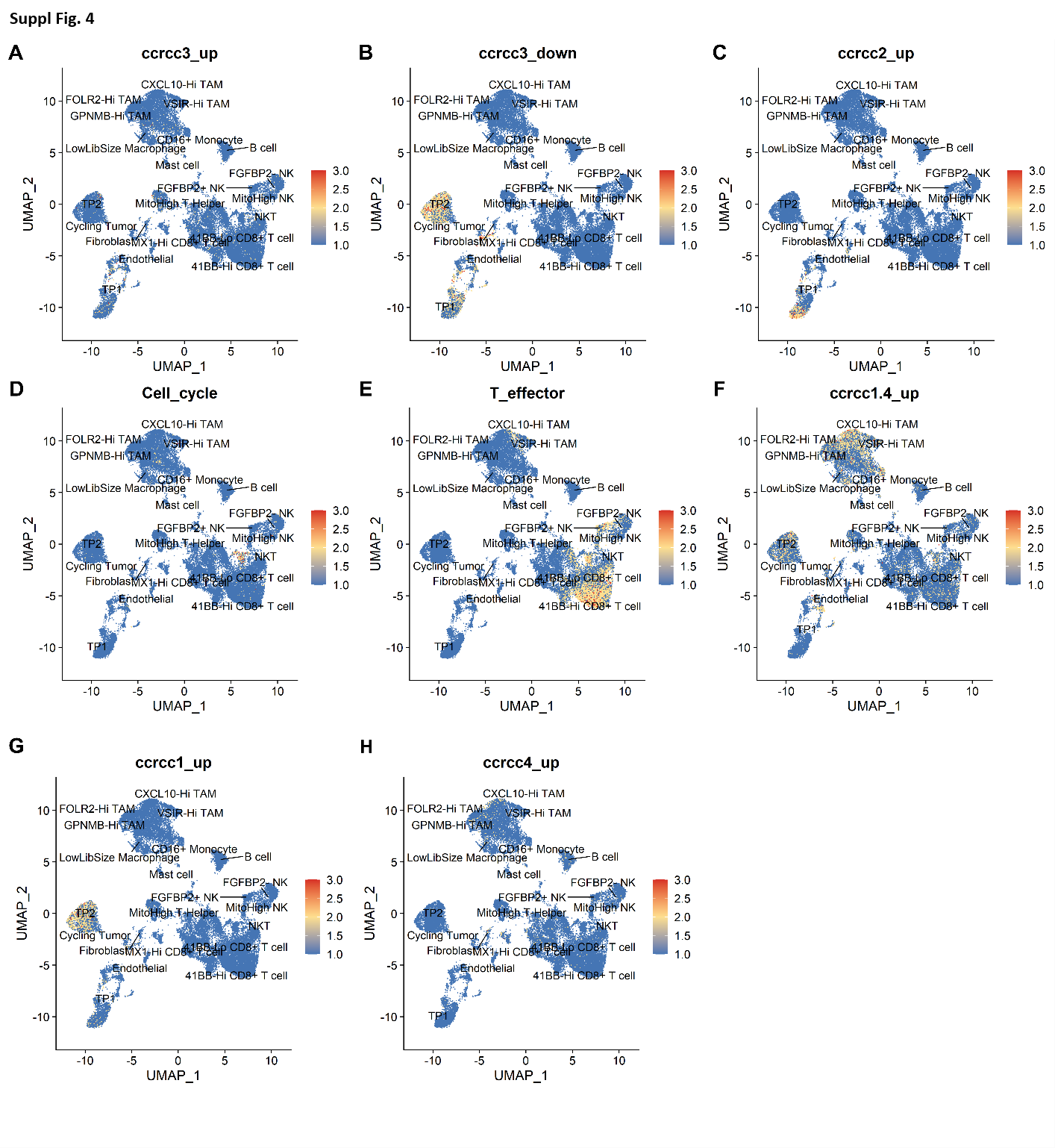


**Suppl. Fig. 4 Single-cell characterization of Clearseq signatures. A-H,** UMAP showing expression of the 8 Clearseq signatures per cell.
