# Supplementary material for: Prognostic and Predictive Value of the Clearseq1–4 Tumor Microenvironment Classification in Localized and Metastatic Clear-Cell Renal Cell Carcinoma
Source: Cancer Res Commun. 2026 Apr 20;6(4):884–97. doi: 10.1158/2767-9764.CRC-25-0548 (PMC13095203; doi:10.1158/2767-9764.CRC-25-0548)
Supplement: Suppl. Figure 5 — Response to angiogenesis inhibitors in later line by Clearseq molecular subtypes [file crc-25-0548_suppl.figure_5_suppsf5.docx]

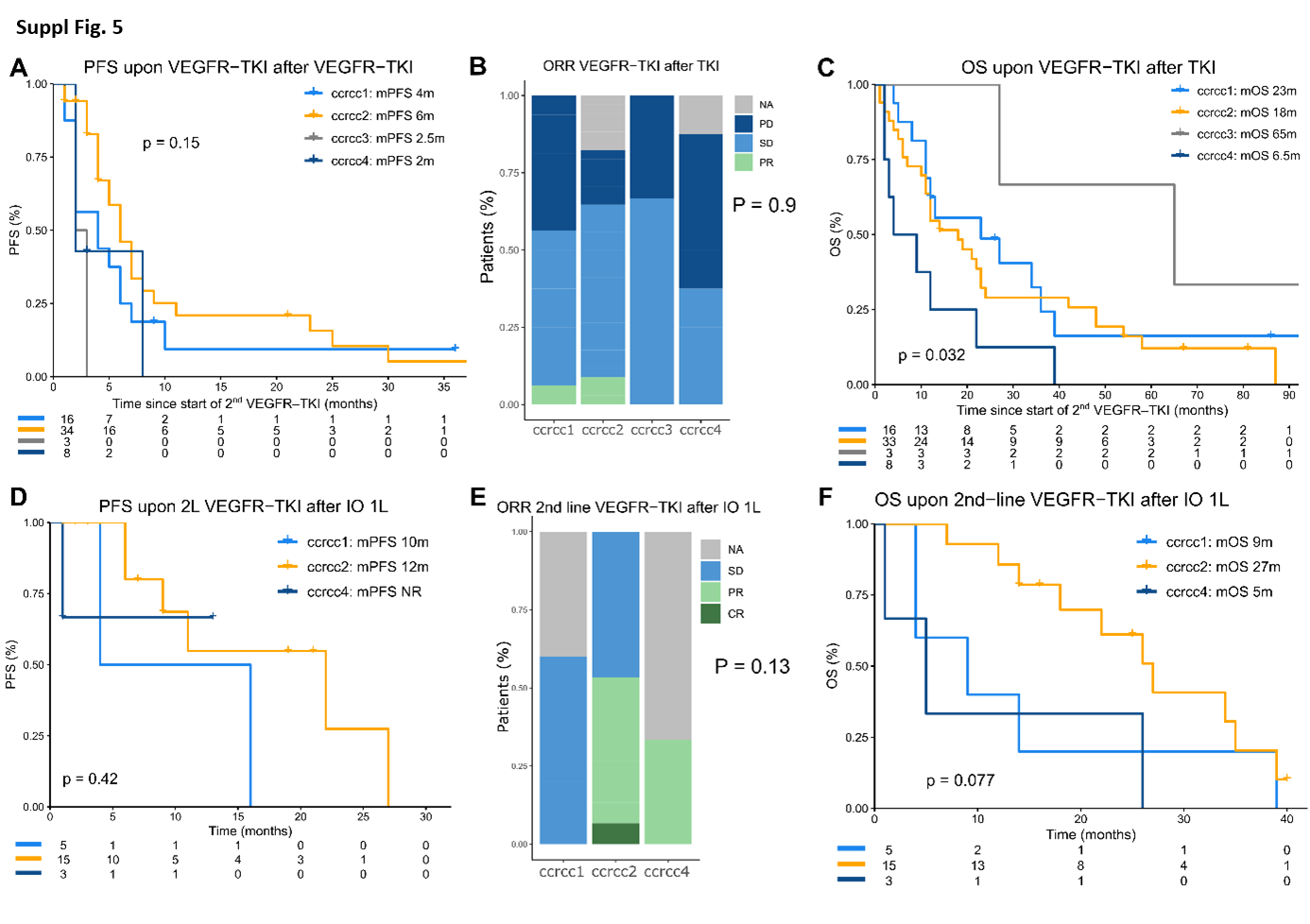


**Suppl. Fig. 5** **Response to angiogenesis inhibitors in later line by Clearseq molecular subtypes. A,** Kaplan-Meier curves showing PFS upon treatment with VEGFR-TKI after VEGFR-TKI by molecular subtype. **B,** Bar chart of best response on VEGFR-TKI after VEGFR-TKI by molecular subtype. **C,** Kaplan-Meier curves showing OS upon VEGFR-TKI after VEGFR-TKI by molecular subtype. **D,** Kaplan-Meier curves showing PFS upon treatment with VEGFR-TKI after ICB as first-line treatment by molecular subtype. **E,** Bar chart of best response on VEGFR-TKI after ICB as first-line treatment by molecular subtype. **F,** Kaplan-Meier curves showing OS upon VEGFR-TKI after ICB as first-line treatment by molecular subtype.
