# Supplementary material for: Prognostic and Predictive Value of the Clearseq1–4 Tumor Microenvironment Classification in Localized and Metastatic Clear-Cell Renal Cell Carcinoma
Source: Cancer Res Commun. 2026 Apr 20;6(4):884–97. doi: 10.1158/2767-9764.CRC-25-0548 (PMC13095203; doi:10.1158/2767-9764.CRC-25-0548)
Supplement: Suppl. Table 1 — Clearseq signatures (A) and comparison with genes included in our previous model (B) based on fresh frozen samples [file crc-25-0548_suppl.table_1_suppst1.docx]

**Suppl. Table 1: Clearseq signatures (A) and comparison with genes included in our previous model (B) based on fresh frozen samples (genes in bold are in common between methods**[**^5^**](https://sciwheel.com/work/citation?ids=7337630&pre=&suf=&sa=0&dbf=0)**).**

| ccrcc3_up | ccrcc3_down | ccrcc2_up | Cell_cycle | T_effector | ccrcc1&4_up | ccrcc4_up | ccrcc1_up |
| --- | --- | --- | --- | --- | --- | --- | --- |
| ANXA9 | **ALDOC** | CD34 | AURKA | **CD274** | FASN | **ADAMDEC1** | ENPP3 |
| CATSPERG | **VEGFA** | ESM1 | BUB1 | **CD38** | PARP1 | **PIM2** | BHMT |
| CDH16 | **CAV1** | FLT1 | BUB1B | CD8A | TALDO1 | **PLAU** | EPO |
| CKMT1A | **TGFA** | PECAM1 | CCNB2 | EOMES | TKT | **SFRP2** | **GRIN2A** |
| KNG1 | **LOX** | **NOSTRIN** | CCNE1 | CXCL10 | PGD | **AIM2** | HABP2 |
| ATP6V0D2 | **PGF** | KDR | CDK2 | CXCL11 | G6PD | **CD274** | **NCAM1** |
| CDH17 | **CP** | FLT4 | MKI67 | GZMA | ACACA | **CD38** | NPTX2 |
| PFKFB2 | **C8orf22** | **LRP2** | POLQ | GZMB | **SLPI** | CD8A | **MAP7D2** |
| SLC22A8 | **ANGPTL4** | **SLC17A4** | CDK4 | PRF1 | **PPP1R1A** | EOMES | SLC28A1 |
| SFRP1 | **CA9** | **SLC5A12** | CDK6 | IFNG | AURKA | CXCL10 | **LRP2** |
| PIGR |  | **SLC6A13** |  | **CXCL13** | BUB1 | CXCL11 | **SULT1C4** |
| PRLR |  | **CUBN** |  | CXCL9 | BUB1B | GZMA | SERPINA3 |
| PVALB |  | **SLC17A3** |  |  | CCNB2 | GZMB | WFDC2 |
| CYP2B6 |  | **GLYAT** |  |  | CCNE1 | PRF1 | HPN |
| ERBB4 |  | **CYP2J2** |  |  | CDK2 | IFNG | KCNK3 |
| DIO1 |  | **ACADL** |  |  | MKI67 | **CXCL13** |  |
| ATP6V0A2 |  | **TNFAIP6** |  |  | POLQ | CXCL9 |  |
|  |  | **SCGN** |  |  | CDK4 |  |  |
|  |  |  |  |  | CDK6 |  |  |

**A**

| ccrcc3_up | ccrcc3_down | ccrcc2_up | ccRCC2_DOWN | CCRCC1_UP | ccrcc1_DOWN | ccrcc4_up | ccrcc4_DOWN |
| --- | --- | --- | --- | --- | --- | --- | --- |
| ANXA9 | **ALDOC** | DAZ1 | A1CF | **C8orf22** | **PIGR** | **ADAMDEC1** | **ACADL** |
| CATSPERG | **VEGFA** | **NOSTRIN** | FGA | EYA1 |  | **PIM2** | **CUBN** |
| CDH16 | **CAV1** | SLC5A1 | **PPP1R1A** | GRIN2A |  | **PLAU** | **GLYAT** |
| CKMT1A | **TGFA** | **CUBN** | **SLPI** | **MAP7D2** |  | **SFRP2** | **LRP2** |
| KNG1 | **LOX** | **SLC17A3** |  | **NCAM1** |  | **AIM2** | NCRNA00262 |
| ATP6V0D2 | **PGF** | **TNFAIP6** |  | SORCS3 |  | **CD274** | **SLC17A3** |
| CDH17 | **CP** | **SCGN** |  | SPOCK1 |  | **CD38** | **SLC6A13** |
| PFKFB2 | **C8orf22** | **CYP2J2** |  | STK39 |  | **SLPI** | **SLC5A12** |
| SLC22A8 | **ANGPTL4** | **SLC17A4** |  |  |  | **CXCL13** | **SULT1C4** |
| SFRP1 | **CA9** | **SLC5A12** |  |  |  | C1QA |  |
| PIGR |  |  |  |  |  | HIST1H3J |  |
| PRLR |  |  |  |  |  | IGHA1 |  |
| PVALB |  |  |  |  |  | IGHD |  |
| CYP2B6 |  |  |  |  |  | IGJ |  |
| ERBB4 |  |  |  |  |  | IGKC |  |
| DIO1 |  |  |  |  |  | IGKV1-5 |  |
| ATP6V0A4 |  |  |  |  |  | KRT19 |  |

**B**
