# Supplementary material for: Prognostic and Predictive Value of the Clearseq1–4 Tumor Microenvironment Classification in Localized and Metastatic Clear-Cell Renal Cell Carcinoma
Source: Cancer Res Commun. 2026 Apr 20;6(4):884–97. doi: 10.1158/2767-9764.CRC-25-0548 (PMC13095203; doi:10.1158/2767-9764.CRC-25-0548)
Supplement: Suppl. Table 2 — Characteristics of patients who received nephrectomy for localised ccRCC [file crc-25-0548_suppl.table_2_suppst2.docx]

| Characteristic | Overall (n=131) | ccrcc1 (n=35) | ccrcc2 (n=72) | ccrcc3 (n=7) | ccrcc4 (n= 17) |
| --- | --- | --- | --- | --- | --- |
| Age at diagnosis (median, interquartile range) | 63 (54-69) | 64 (53-70) | 63 (55-68) | 52 (45-61) | 62 (61-71) |
| Sex: female (%) | 44 (34%) | 15 (43%) | 21 (29%) | 2 (29%) | 6 (35%) |
| Fuhrman grade - no. (%) |  |  |  |  |  |
| * Grade I | 0 (0%) | 0 (0%) | 0 (0%) | 0 (0%) | 0 (0%) |
| * Grade II | 12 (9.2%) | 4 (11%) | 6 (8.3%) | 0 (0%) | 2 (12%) |
| * Grade III | 57 (44%) | 10 (29%) | 38 (53%) | 4 (57%) | 5 (29%) |
| * Grade IV | 62 (47%) | 21 (60%) | 28 (39%) | 3 (43%) | 10 (59%) |
| T |  |  |  |  |  |
| * 1a/b | 36 (27%) | 5 (14%) | 25 (35%) | 2 (29%) | 4 (24%) |
| * 2a/b | 29 (22%) | 7 (20%) | 17 (24%) | 3 (43%) | 2 (12%) |
| * 3a/b/c | 58 (44%) | 23 (66%) | 22 (31%) | 2 (29%) | 11 (65%) |
| * 4 | 3 (2.3%) | 0 (0%) | 3 (4.2%) | 0 (0%) | 0 (0%) |
| * Unknown | 5 (3.8%) | 0 (0%) | 5 (6.9%) | 0 (0%) | 0 (0%) |
| N |  |  |  |  |  |
| * 0 | 79 (60%) | 25 (71%) | 37 (51%) | 5 (71%) | 12 (71%) |
| * 1 | 5 (3.8%) | 1 (2.9%) | 2 (2.8%) | 1 (14%) | 1 (5.9%) |
| * 2 | 1 (0.8%) | 0 (0%) | 1 (1.4%) | 0 (0%) | 0 (0%) |
| * Unknown | 46 (35%) | 9 (26%) | 32 (44%) | 1 (14%) | 4 (24%) |
| Sarcomatoid differentiation (mean %, SD) | 14 (102) | 12 (56) | 2 (11) | 0 (0) | 70 (257) |
| Treated with ICB before death (n, %) | 60 (46%) | 18 (51%) | 32 (44%) | 3 (43%) | 7 (41%) |

**Suppl. Table 2: Characteristics of patients who received nephrectomy for localised ccRCC**
