# Supplementary material for: Prognostic and Predictive Value of the Clearseq1–4 Tumor Microenvironment Classification in Localized and Metastatic Clear-Cell Renal Cell Carcinoma
Source: Cancer Res Commun. 2026 Apr 20;6(4):884–97. doi: 10.1158/2767-9764.CRC-25-0548 (PMC13095203; doi:10.1158/2767-9764.CRC-25-0548)
Supplement: Suppl. Table 3 — Multivariable models for nephrectomy for localised ccRCC and debulking nephrectomy [file crc-25-0548_suppl.table_3_suppst3.docx]

|  | Nephrectomy for localised ccRCC | | | | | | Debulking nephrectomy | | | | | | |
| --- | --- | --- | --- | --- | --- | --- | --- | --- | --- | --- | --- | --- | --- |
|  | **HR (DFS)** | **95% CI** | **p-value** | **HR (OS)** | **95% CI** | **p-value** | | **HR (STFS)** | **95% CI** | **p-value** | **HR (OS)** | **95% CI** | **p-value** |
| Clearseq |  |  |  |  |  |  | |  |  |  |  |  |  |
| * ccrcc1 | — | — | — | — | — | — | | — | — | — | — | — | — |
| * ccrcc2 | 0.62 | 0.40, 0.97 | 0.038 | 0.75 | 0.41, 1.35 | 0.3 | | 0.77 | 0.44, 1.33 | 0.3 | 0.71 | 0.40, 1.25 | 0.2 |
| * ccrcc3 | 0.9 | 0.37, 2.14 | 0.8 | 1.6 | 0.57, 4.48 | 0.4 | | 1.43 | 0.41, 5.00 | 0.6 | 2.24 | 0.64, 7.84 | 0.2 |
| * ccrcc4 | 1.25 | 0.69, 2.26 | 0.5 | 2.4 | 1.17, 4.92 | 0.017 | | 2.12 | 1.11, 4.02 | 0.022 | 1.37 | 0.68, 2.76 | 0.4 |
| T |  |  |  |  |  |  | |  |  |  |  |  |  |
| * 1a/b | — | — | — | — | — | — | | — | — | — | — | — | — |
| * 2a/b | 0.97 | 0.57, 1.67 | >0.9 | 0.76 | 0.39, 1.48 | 0.4 | | 1.23 | 0.49, 3.09 | 0.7 | 0.79 | 0.27, 2.31 | 0.7 |
| * 3a/b/c | 1.52 | 0.95, 2.45 | 0.084 | 1.31 | 0.74, 2.32 | 0.4 | | 1.54 | 0.78, 3.03 | 0.2 | 1.25 | 0.62, 2.53 | 0.5 |
| * 4 | 0.99 | 0.29, 3.36 | >0.9 | 3.44 | 0.78, 15.2 | 0.1 | | 2.24 | 0.92, 5.47 | 0.076 | 1.52 | 0.57, 4.06 | 0.4 |
| * UNKNOWN | 0.61 | 0.22, 1.66 | 0.3 | 2.05 | 0.70, 6.02 | 0.2 | | 0.62 | 0.13, 2.86 | 0.5 | 0.39 | 0.05, 3.14 | 0.4 |
| N |  |  |  |  |  |  | |  |  |  |  |  |  |
| * 0 | — | — | — | — | — | — | | — | — | — | — | — | — |
| * 1 | 0.95 | 0.33, 2.72 | >0.9 | 0.2 | 0.05, 0.73 | 0.015 | | 2.06 | 1.23, 3.44 | 0.006 | 1.58 | 0.94, 2.66 | 0.087 |
| * 2 | 1.67 | 0.22, 12.6 | 0.6 | 0 | 0.00, Inf | >0.9 | | / | / | / | / | / | / |
| * UNKNOWN | 1.14 | 0.75, 1.74 | 0.5 | 0.48 | 0.27, 0.84 | 0.01 | | 1.6 | 0.92, 2.78 | 0.1 | 1.41 | 0.77, 2.60 | 0.3 |
| Fuhrman grade |  |  |  |  |  |  | |  |  |  |  |  |  |
| * 1 | / | / | / | / | / | / | | — | — | — | — | — | — |
| * 2 | — | — | — | — | — | — | | 1.59 | 0.18, 14.2 | 0.7 | 0.76 | 0.08, 7.14 | 0.8 |
| * 3 | 1.53 | 0.76, 3.05 | 0.2 | 3.13 | 1.18, 8.30 | 0.022 | | 0.66 | 0.08, 5.13 | 0.7 | 0.52 | 0.07, 4.06 | 0.5 |
| * 4 | 1.91 | 0.96, 3.78 | 0.064 | 4.82 | 1.84, 12.7 | 0.001 | | 0.96 | 0.13, 7.42 | >0.9 | 0.65 | 0.08, 5.07 | 0.7 |
| HR = Hazard Ratio, CI = Confidence Interval, DFS = disease-free survival, STFS = systemic therapy free survival, OS = overall survival | | | | | | | | | | | | | |

**Suppl. Table 3: Multivariable models for nephrectomy for localised ccRCC and debulking nephrectomy**
