# Supplementary material for: Prognostic and Predictive Value of the Clearseq1–4 Tumor Microenvironment Classification in Localized and Metastatic Clear-Cell Renal Cell Carcinoma
Source: Cancer Res Commun. 2026 Apr 20;6(4):884–97. doi: 10.1158/2767-9764.CRC-25-0548 (PMC13095203; doi:10.1158/2767-9764.CRC-25-0548)
Supplement: Suppl. Table 4 — Characteristics of patients who received debulking [file crc-25-0548_suppl.table_4_suppst4.docx]

| Characteristic | Overall (n=102) | ccrcc1 (n=23) | ccrcc2 (n=88) | ccrcc3 (n=9) | ccrcc4 (n= 29) |
| --- | --- | --- | --- | --- | --- |
| Age at diagnosis (median, interquartile range) | 62 (54-67) | 59 (51-66) | 62 (56-68) | 63 (62-66) | 61 (52-64) |
| Sex: female (%) | 27 (26%) | 5 (22%) | 14 (25%) | 1 (33%) | 7 (37%) |
| Fuhrman grade - no. (%) |  |  |  |  |  |
| * Grade I | 1 (1%) | 0 (0%) | 1 (1.8%) | 0 (0%) | 0 (0%) |
| * Grade II | 6 (5.9%) | 0 (0%) | 6 (11%) | 0 (0%) | 0 (0%) |
| * Grade III | 25 (25%) | 6 (26%) | 18 (32%) | 0 (0%) | 1 (5.3%) |
| * Grade IV | 70 (69%) | 17 (74%) | 32 (56%) | 3 (100%) | 18 (95%) |
| T |  |  |  |  |  |
| * 1a/b | 13 (13%) | 2 (8.7%) | 11 (19%) | 0 (0%) | 0 (0%) |
| * 2a/b | 8 (7.8%) | 2 (8.7%) | 5 (8.8%) | 0 (0%) | 1 (5.3%) |
| * 3a/b/c | 69 (68%) | 16 (70%) | 34 (60%) | 3 (100%) | 16 (84%) |
| * 4 | 10 (9.8%) | 3 (13%) | 5 (8.8%) | 0 (0%) | 2 (11%) |
| * Unknown | 2 (2.0%) | 0 (0%) | 2 (3.5%) | 0 (0%) | 0 (0%) |
| N |  |  |  |  |  |
| * 0 | 44 (43%) | 11 (48%) | 26 (46%) | 1 (33%) | 6 (32%) |
| * 1 | 32 (31%) | 8 (35%) | 12 (21%) | 2 (67%) | 10 (53%) |
| * Unknown | 26 (25%) | 4 (17%) | 19 (33%) | 0 (0%) | 3 (16%) |
| Sarcomatoid differentiation (mean %, SD) | 6 (18) | 2 (5) | 3 (15) | 2 (3) | 22 (29) |
| Treated with ICB before death (n, %) | 51 (50%) | 12 (52%) | 33 (58%) | 0 (0%) | 6 (32%) |

**Suppl. Table 4: Characteristics of patients who received debulking nephrectomy**
