# Supplementary material for: Prognostic and Predictive Value of the Clearseq1–4 Tumor Microenvironment Classification in Localized and Metastatic Clear-Cell Renal Cell Carcinoma
Source: Cancer Res Commun. 2026 Apr 20;6(4):884–97. doi: 10.1158/2767-9764.CRC-25-0548 (PMC13095203; doi:10.1158/2767-9764.CRC-25-0548)
Supplement: Suppl. Table 5 — Characteristics of patients who received metastasectomy with curative intent [file crc-25-0548_suppl.table_5_suppst5.docx]

| Characteristic | Overall (n=72) | ccrcc1 (n=20) | ccrcc2 (n=41) | ccrcc3 (n=3) | ccrcc4 (n= 8) |
| --- | --- | --- | --- | --- | --- |
| Age at nephrectomy (median, interquartile range) | 61 (53, 66) | 56 (50, 65) | 61 (53, 66) | 50 (48, 55) | 66 (62, 72) |
| Sex: female (%) | 22 (31%) | 10 (50%) | 7 (17%) | 2 (67%) | 3 (38%) |
| Fuhrman grade - no. (%) |  |  |  |  |  |
| * Grade II | 13 (18%) | 4 (20%) | 8 (20%) | 0 (0%) | 1 (12%) |
| * Grade III | 27 (38%) | 4 (20%) | 18 (44%) | 2 (67%) | 3 (38%) |
| * Grade IV | 30 (42%) | 12 (60%) | 14 (34%) | 1 (33%) | 3 (38%) |
| * Unknown | 2 (2.8%) | 0 (0%) | 1 (2.4%) | 0 (0%) | 1 (12%) |
| T |  |  |  |  |  |
| * 1a/b | 16 (22%) | 3 (15%) | 10 (24%) | 0 (0%) | 3 (38%) |
| * 2a/b | 18 (25%) | 4 (20%) | 12 (29%) | 1 (33%) | 1 (12%) |
| * 3a/b/c | 35 (49%) | 13 (65%) | 16 (39%) | 2 (67%) | 4 (50%) |
| * 4 | 1 (1.4%) | 0 (0%) | 1 (2.4%) | 0 (0%) | 0 (0%) |
| * Unknown | 2 (2.8%) | 0 (0%) | 2 (4.9%) | 0 (0%) | 0 (0%) |
| N |  |  |  |  |  |
| * 0 | 37 (51%) | 11 (55%) | 17 (41%) | 2 (67%) | 7 (88%) |
| * 1 | 2 (2.8%) | 1 (5.0%) | 1 (2.4%) | 0 (0%) | 0 (0%) |
| * Unknown | 33 (46%) | 8 (40%) | 23 (56%) | 1 (33%) | 1 (12%) |
| Sarcomatoid differentiation (mean %, SD) | 0.49 (1.70) | 0.50 (1.32) | 0.07 (0.26) | 0.00 (0.00) | 3.00 (4.47) |
| Treated with ICB before death (n, %) | 28 (39%) | 10 (50%) | 15 (37%) | 1 (33%) | 2 (25%) |
| Leuven Udine - no. (%) |  |  |  |  |  |
| * A | 13 (19%) | 2 (10%) | 9 (24%) | 0 (0%) | 2 (29%) |
| * B | 20 (29%) | 8 (40%) | 11 (29%) | 0 (0%) | 1 (14%) |
| * C | 26 (38%) | 6 (30%) | 16 (42%) | 3 (100%) | 1 (14%) |
| * D | 9 (13%) | 4 (20%) | 2 (5.3%) | 0 (0%) | 3 (43%) |
| * Unknown | 4 | 0 | 3 | 0 | 1 |
| Metachronous metastases (vs. synchr.) - no. (%) | 60 (83%) | 19 (95%) | 32 (78%) | 2 (67%) | 7 (88%) |

**Suppl. Table 5: Characteristics of patients who received metastasectomy with curative intent**
