# Supplementary material for: Prognostic and Predictive Value of the Clearseq1–4 Tumor Microenvironment Classification in Localized and Metastatic Clear-Cell Renal Cell Carcinoma
Source: Cancer Res Commun. 2026 Apr 20;6(4):884–97. doi: 10.1158/2767-9764.CRC-25-0548 (PMC13095203; doi:10.1158/2767-9764.CRC-25-0548)
Supplement: Suppl. Table 6 — Multivariable models within metastasectomy with curative intent cohort [file crc-25-0548_suppl.table_6_suppst6.docx]

|  | HR DFS (95% CI) | p-value | HR STFS (95% CI) | p-value | HR OS (95% CI) | p-value |
| --- | --- | --- | --- | --- | --- | --- |
| Clearseq |  |  |  |  |  |  |
| * ccrcc1 | — | — | — | — | — | — |
| * ccrcc2 | 0.51 (0.27, 0.94) | 0.032 | 0.61 (0.31, 1.21) | 0.2 | 0.77 (0.32, 1.83) | 0.6 |
| * ccrcc3 | 1 (0.27, 3.72) | >0.9 | 2.15 (0.56, 8.32) | 0.3 | 1.22 (0.23, 6.34) | 0.8 |
| * ccrcc4 | 1.28 (0.52, 3.17) | 0.6 | 1.39 (0.56, 3.48) | 0.5 | 3.12 (1.06, 9.20) | 0.039 |
| Leuven Udine |  |  |  |  |  |  |
| * A | — | — | — | — | — | — |
| * B | 1.04 (0.48, 2.26) | >0.9 | 1.45 (0.62, 3.39) | 0.4 | 0.92 (0.31, 2.77) | 0.9 |
| * C | 1.38 (0.64, 2.98) | 0.4 | 1.58 (0.69, 3.64) | 0.3 | 2.87 (1.05, 7.85) | 0.04 |
| * D | 5.39 (2.10, 13.8) | <0.001 | 10.4 (3.48, 31.1) | <0.001 | 15.7 (4.73, 52.1) | <0.001 |

**Suppl. Table 6: Multivariable models within metastasectomy with curative intent cohort**
