# Supplementary material for: Prognostic and Predictive Value of the Clearseq1–4 Tumor Microenvironment Classification in Localized and Metastatic Clear-Cell Renal Cell Carcinoma
Source: Cancer Res Commun. 2026 Apr 20;6(4):884–97. doi: 10.1158/2767-9764.CRC-25-0548 (PMC13095203; doi:10.1158/2767-9764.CRC-25-0548)
Supplement: Suppl. Table 7 — Patient characteristics of the VEGFR-TKI in first-line cohort [file crc-25-0548_suppl.table_7_suppst7.docx]

**Suppl. Table 7: Patient characteristics of the VEGFR-TKI in first-line cohort**

| Characteristic | Overall (n=170) | ccrcc1 (n=44) | ccrcc2 (n=88) | ccrcc3 (n=9) | ccrcc4 (n= 29) |
| --- | --- | --- | --- | --- | --- |
| Age at diagnosis (median, interquartile range) | 62 (55, 68) | 63 (54, 70) | 62 (56,68) | 58 (45, 62) | 62 (56, 69) |
| Age at start of therapy (median, interquartile range) | 65 (59,72) | 65 (58,72) | 66 (61,73) | 62 (53,66) | 64 (59,73) |
| Sex: female (%) | 54 (32%) | 17 (39%) | 23 (26%) | 3 (33%) | 11 (38%) |
| Fuhrman grade - no. (%) |  |  |  |  |  |
| * Grade I | 1 (0.6%) | 0 (0%) | 1 (1.1%) | 0 (0%) | 0 (0%) |
| * Grade II | 14 (8.2%) | 3 (6.8%) | 8 (9.1%) | 0 (0%) | 3 (10%) |
| * Grade III | 58 (34%) | 15 (34%) | 35 (40%) | 4 (44%) | 4 (14%) |
| * Grade IV | 97 (57%) | 26 (59%) | 44 (50%) | 5 (56%) | 22 (76%) |
| IMDC - no. (%) |  |  |  |  |  |
| * Good risk | 33 (19%) | 8 (18%) | 19 (22%) | 2 (22%) | 4 (14%) |
| * Intermediate risk | 96 (56%) | 29 (66%) | 54 (61%) | 5 (56%) | 8 (28%) |
| * Poor risk | 38 (22%) | 7 (16%) | 14 (16%) | 2 (22%) | 15 (52%) |
| * Unknown | 3 (1.8%) | 0 (0%) | 1 (1.1%) | 0 (0%) | 2 (6.9%) |
| Type of VEGFR-TKI - no. (%) |  |  |  |  |  |
| * Pazopanib | 71 (42%) | 19 (43%) | 34 (39%) | 4 (44%) | 14 (48%) |
| * Sorafenib | 9 (5.3%) | 3 (6.8%) | 4 (4.5%) | 1 (11%) | 1 (3.4%) |
| * Sunitinib | 90 (53%) | 22 (50%) | 50 (57%) | 4 (44%) | 14 (48%) |
| ICB in later line - no. (%) | 78 (46%) | 22 (50%) | 44 (50%) | 3 (33%) | 9 (31%) |
| Sarcomatoid differentiation (mean, standard deviation) | 4% (14%) | 1% (5%) | 2% (13%) | 0% (0%) | 12% (23%) |
