# Supplementary material for: Prognostic and Predictive Value of the Clearseq1–4 Tumor Microenvironment Classification in Localized and Metastatic Clear-Cell Renal Cell Carcinoma
Source: Cancer Res Commun. 2026 Apr 20;6(4):884–97. doi: 10.1158/2767-9764.CRC-25-0548 (PMC13095203; doi:10.1158/2767-9764.CRC-25-0548)
Supplement: Suppl. Table 8 — Multivariable cox proportional hazards models [file crc-25-0548_suppl.table_8_suppst8.docx]

**Suppl. Table 8: Multivariable cox proportional hazards models**

|  | VEGFR-TKI first line | | | | VEGFR-TKI after VEGFR-TKI | | | |  |
| --- | --- | --- | --- | --- | --- | --- | --- | --- | --- |
|  | HR PFS (95% CI) | P | HR OS (95% CI) | P | HR PFS (95% CI) | P | HR OS (95% CI) | P | |
| Clearseq |  |  |  |  |  |  |  |  | |
| * ccrcc1 | — | — | — | — | — | — | — | — | |
| * ccrcc2 | 0.69 (0.46, 1.05) | 0.087 | 1.09 (0.72, 1.66) | 0.7 | 0.75 (0.36, 1.54) | 0.4 | 1.26 (0.61, 2.62) | 0.5 | |
| * ccrcc3 | 0.85 (0.39, 1.85) | 0.7 | 0.83 (0.37, 1.89) | 0.7 | 4.54 (0.93, 22.3) | 0.062 | 0.38 (0.08, 1.89) | 0.2 | |
| * ccrcc4 | 1.89 (1.13, 3.18) | 0.016 | 1.63 (0.94, 2.84) | 0.082 | 1.72 (0.59, 5.02) | 0.3 | 3.89 (1.41, 10.7) | 0.009 | |
| IMDC |  |  |  |  |  |  |  |  | |
| * GOOD | — | — | — | — | — | — | — | — | |
| * INTERMEDIATE | 2.12 (1.32, 3.42) | 0.002 | 2.5 (1.52, 4.13) | <0.001 | 5.64 (0.76, 41.9) | 0.091 | 5.23 (0.70, 39.1) | 0.11 | |
| * POOR | 4.26 (2.46, 7.40) | <0.001 | 5.58 (3.10, 10.1) | <0.001 | 6.3 (0.80, 49.8) | 0.081 | 9.62 (1.24, 74.6) | 0.03 | |
