# Supplementary material for: Prognostic and Predictive Value of the Clearseq1–4 Tumor Microenvironment Classification in Localized and Metastatic Clear-Cell Renal Cell Carcinoma
Source: Cancer Res Commun. 2026 Apr 20;6(4):884–97. doi: 10.1158/2767-9764.CRC-25-0548 (PMC13095203; doi:10.1158/2767-9764.CRC-25-0548)
Supplement: Suppl. Table 9 — Patient characteristics in the sequential TKI cohort [file crc-25-0548_suppl.table_9_suppst9.docx]

**Suppl. Table 9: Patient characteristics in the sequential TKI cohort**

| Characteristic | Overall, N = 61 | ccrcc1, N = 16 | ccrcc2, N = 34 | ccrcc3, N = 3 | ccrcc4, N = 8 |
| --- | --- | --- | --- | --- | --- |
| Age at diagnosis (median, interquartile range) | 59 (52, 66) | 60 (52, 65) | 60 (54, 68) | 52 (48, 56) | 56 (51, 60) |
| Sex: female (%) | 16 (26%) | 6 (38%) | 6 (18%) | 1 (33%) | 3 (38%) |
| Fuhrman grade - no. (%) |  |  |  |  |  |
| * Grade I | 0 (0%) | 0 (0%) | 0 (0%) | 0 (0%) | 0 (0%) |
| * Grade II | 9 (15%) | 2 (12%) | 7 (21%) | 0 (0%) | 0 (0%) |
| * Grade III | 19 (31%) | 5 (31%) | 11 (32%) | 2 (67%) | 1 (12%) |
| * Grade IV | 33 (54%) | 9 (56%) | 16 (47%) | 1 (33%) | 7 (88%) |
| IMDC - no. (%) |  |  |  |  |  |
| * Good risk | 4 (6.6%) | 1 (6.2%) | 2 (5.9%) | 1 (33%) | 0 (0%) |
| * Intermediate risk | 37 (61%) | 10 (62%) | 24 (71%) | 1 (33%) | 2 (25%) |
| * Poor risk | 17 (28%) | 3 (19%) | 8 (24%) | 1 (33%) | 5 (62%) |
| * Unknown | 3 (4.9%) | 2 (12%) | 0 (0%) | 0 (0%) | 1 (12%) |
| Line of sequential TKI |  |  |  |  |  |
| * First and second line | 45 (74%) | 13 (81%) | 24 (71%) | 3 (100%) | 5 (62%) |
| * Second and third line | 14 (23%) | 3 (19%) | 9 (26%) | 0 (0%) | 2 (25%) |
| * Third and fourth line | 8 (13%) | 2 (12%) | 5 (15%) | 0 (0%) | 1 (12%) |
| * Fourth and fifth line | 1 (1.6%) | 0 (0%) | 1 (2.9%) | 0 (0%) | 0 (0%) |
| * Fifth and sixth line | 1 (1.6%) | 0 (0%) | 1 (2.9%) | 0 (0%) | 0 (0%) |
| * Sixth and seventh line | 1 (1.6%) | 1 (6.2%) | 0 (0%) | 0 (0%) | 0 (0%) |
|  | | | | | |
