# Supplementary material for: Prognostic and Predictive Value of the Clearseq1–4 Tumor Microenvironment Classification in Localized and Metastatic Clear-Cell Renal Cell Carcinoma
Source: Cancer Res Commun. 2026 Apr 20;6(4):884–97. doi: 10.1158/2767-9764.CRC-25-0548 (PMC13095203; doi:10.1158/2767-9764.CRC-25-0548)
Supplement: Suppl. Table 10 — Patient characteristics for the VEGFR-TKI after ICB cohort [file crc-25-0548_suppl.table_10_suppst10.docx]

| Characteristic | Overall, N = 23 | ccrcc1, N = 5 | ccrcc2, N = 15 | ccrcc4, N = 3 |
| --- | --- | --- | --- | --- |
| Age at diagnosis (median, interquartile range) | 62 (51, 66) | 51 (50, 63) | 62 (52, 66) | 66 (64, 68) |
| Sex: female (%) | 5 (22%) | 0 (0%) | 4 (27%) | 1 (33%) |
| Fuhrman grade - no. (%) |  |  |  |  |
| * Grade I | 0 (0%) | 0 (0%) | 0 (0%) | 0 (0%) |
| * Grade II | 2 (8.7%) | 0 (0%) | 2 (13%) | 0 (0%) |
| * Grade III | 7 (30%) | 0 (0%) | 7 (47%) | 0 (0%) |
| * Grade IV | 14 (61%) | 5 (100%) | 6 (40%) | 3 (100%) |
| IMDC - no. (%) |  |  |  |  |
| * Good risk | 2 (8.7%) | 0 (0%) | 2 (13%) | 0 (0%) |
| * Intermediate risk | 15 (65%) | 4 (80%) | 10 (67%) | 1 (33%) |
| * Poor risk | 6 (26%) | 1 (20%) | 3 (20%) | 2 (67%) |
| Type of TKI in 2nd line - no. (%) |  |  |  |  |
| * AXITINIB | 6 (26%) | 2 (40%) | 3 (20%) | 1 (33%) |
| * CABOZANTINIB | 12 (52%) | 1 (20%) | 9 (60%) | 2 (67%) |
| * SUNITINIB | 5 (22%) | 2 (40%) | 3 (20%) | 0 (0%) |
| First line treatment - no. (%) |  |  |  |  |
| * AVELUMAB | 1 (4.3%) | 1 (20%) | 0 (0%) | 0 (0%) |
| * AXITINIB/PEMBROLIZUMAB | 1 (4.3%) | 0 (0%) | 0 (0%) | 1 (33%) |
| * IPILIMUMAB/NIVOLUMAB | 20 (87%) | 3 (60%) | 15 (100%) | 2 (67%) |
| * NIVOLUMAB | 1 (4.3%) | 1 (20%) | 0 (0%) | 0 (0%) |

**Suppl. Table 10: Patient characteristics for the VEGFR-TKI after ICB cohort**
