# Supplementary material for: Prognostic and Predictive Value of the Clearseq1–4 Tumor Microenvironment Classification in Localized and Metastatic Clear-Cell Renal Cell Carcinoma
Source: Cancer Res Commun. 2026 Apr 20;6(4):884–97. doi: 10.1158/2767-9764.CRC-25-0548 (PMC13095203; doi:10.1158/2767-9764.CRC-25-0548)
Supplement: Suppl. Table 11 — Patient characteristics of the dual ICB cohort [file crc-25-0548_suppl.table_11_suppst11.docx]

**Suppl. Table 11: Patient characteristics of the dual ICB cohort**

| Characteristic | Overall, N = 36 | ccrcc1, N = 8 | ccrcc2, N = 23 | ccrcc4, N = 5 |
| --- | --- | --- | --- | --- |
| Age at diagnosis (median, interquartile range) | 56 (50, 66) | 58 (49, 69) | 57 (50, 66) | 56 (51, 62) |
| Age at start of ICB (median, interquartile range) | 64 (52, 70) | 66 (50, 71) | 64 (53, 69) | 56 (52, 64) |
| Sex: female (%) | 8 (22%) | 1 (12%) | 6 (26%) | 1 (20%) |
| Fuhrman grade - no. (%) |  |  |  |  |
| * Grade I |  |  |  |  |
| * Grade II | 3 (8.3%) | 1 (12%) | 2 (8.7%) | 0 (0%) |
| * Grade III | 12 (33%) | 0 (0%) | 11 (48%) | 1 (20%) |
| * Grade IV | 21 (58%) | 7 (88%) | 10 (43%) | 4 (80%) |
| IMDC - no. (%) |  |  |  |  |
| * Good risk | 9 (25%) | 1 (12%) | 6 (26%) | 2 (40%) |
| * Intermediate risk | 24 (67%) | 6 (75%) | 17 (74%) | 1 (20%) |
| * Poor risk | 3 (8.3%) | 1 (12%) | 0 (0%) | 2 (40%) |
