# Supplementary material for: Prognostic and Predictive Value of the Clearseq1–4 Tumor Microenvironment Classification in Localized and Metastatic Clear-Cell Renal Cell Carcinoma
Source: Cancer Res Commun. 2026 Apr 20;6(4):884–97. doi: 10.1158/2767-9764.CRC-25-0548 (PMC13095203; doi:10.1158/2767-9764.CRC-25-0548)
Supplement: Suppl. Table 12 — Patient characteristics of the cohort treated with nivolumab in later line [file crc-25-0548_suppl.table_12_suppst12.docx]

| Characteristic | Overall (n=82) | ccrcc1 (n=24) | ccrcc2 (n=45) | ccrcc3 (n=3) | ccrcc4 (n= 10) |
| --- | --- | --- | --- | --- | --- |
| Age at diagnosis (median, interquartile range) | 61 (54-68) | 62 (52-70) | 61 (55-67) | 52 (48-56) | 62 (56-68) |
| Age at start of therapy (median, interquartile range) | 66 (59-74) | 66 (60-72) | 66 (60-75) | 57 (56-61) | 66 (62-79) |
| Sex: female (%) | 22 (27%) | 6 (25%) | 12 (27%) | 1 (33%) | 3 (30%) |
| Fuhrman grade - no. (%) |  |  |  |  |  |
| * Grade I | 1 (1.2%) | 0 (0%) | 1 (2.2%) | 0 (0%) | 0 (0%) |
| * Grade II | 12 (15%) | 3 (12%) | 7 (16%) | 0 (0%) | 2 (20%) |
| * Grade III | 31 (38%) | 12 (50%) | 14 (31%) | 2 (67%) | 3 (30%) |
| * Grade IV | 38 (46%) | 9 (38%) | 23 (51%) | 1 (33%) | 5 (50%) |
| IMDC - no. (%) |  |  |  |  |  |
| * Good risk | 9 (11%) | 3 (12%) | 5 (11%) | 1 (33%) | 0 (0%) |
| * Intermediate risk | 49 (60%) | 15 (62%) | 25 (56%) | 2 (67%) | 7 (70%) |
| * Poor risk | 23 (28%) | 5 (21%) | 15 (33%) | 0 (0%) | 3 (30%) |
| * Unknown | 1 (1.2%) | 1 (4.2%) | 0 (0%) | 0 (0%) | 0 (0%) |
| Line of ICB - no. (%) |  |  |  |  |  |
| * First line | 2 (2.4%) | 1 (4.2%) | 1 (2.2%) | 0 (0%) | 0 (0%) |
| * Second line | 51 (62%) | 15 (62%) | 30 (67%) | 0 (0%) | 6 (60%) |
| * Third line | 22 (27%) | 5 (21%) | 12 (27%) | 2 (67%) | 3 (30%) |
| * Fourth line | 5 (6.1%) | 1 (4.2%) | 2 (4.4%) | 1 (33%) | 1 (10%) |
| * Beyond fourth line | 2 (2.4%) | 2 (8.3%) | 0 (0%) | 0 (0%) | 0 (0%) |
| Previous VEGFR-TKI treatment - no. (%) |  |  |  |  |  |
| * Pazopanib | 50 (61%) | 14 (58%) | 27 (60%) | 2 (67%) | 7 (70%) |
| * Sorafenib | 4 (4.9%) | 2 (8.3%) | 1 (2.2%) | 0 (0%) | 1 (10%) |
| * Sunitinib | 32 (39%) | 9 (38%) | 19 (42%) | 1 (33%) | 3 (30%) |
| * Axitinib | 25 (30%) | 8 (33%) | 11 (24%) | 3 (100%) | 3 (30%) |
| * Everolimus | 6 (7.3%) | 3 (12%) | 1 (2.2%) | 1 (33%) | 1 (10%) |

**Suppl. Table 12: Patient characteristics of the cohort treated with nivolumab in later line**
