# Supplementary material for: Prognostic and Predictive Value of the Clearseq1–4 Tumor Microenvironment Classification in Localized and Metastatic Clear-Cell Renal Cell Carcinoma
Source: Cancer Res Commun. 2026 Apr 20;6(4):884–97. doi: 10.1158/2767-9764.CRC-25-0548 (PMC13095203; doi:10.1158/2767-9764.CRC-25-0548)
Supplement: Suppl. Table 13 — Bivariable cox proportional hazards models [file crc-25-0548_suppl.table_13_suppst13.docx]

**Suppl. Table 13: Bivariable cox proportional hazards models**

|  | Nivolumab in later line | | | |
| --- | --- | --- | --- | --- |
|  | HR PFS (95% CI) | P | HR OS (95% CI) | P |
| Clearseq |  |  |  |  |
| * ccrcc1 | — | — | — | — |
| * ccrcc2 | 0.81 (0.46, 1.41) | 0.5 | 1.49 (0.78, 2.86) | 0.2 |
| * ccrcc3 | 1.37 (0.40, 4.68) | 0.6 | 0.94 (0.21, 4.27) | >0.9 |
| * ccrcc4 | 0.92 (0.40, 2.10) | 0.8 | 1.69 (0.69, 4.12) | 0.3 |
| IMDC |  |  |  |  |
| * GOOD | — | — | — | — |
| * INTERMEDIATE | 0.88 (0.41, 1.90) | 0.7 | 1.01 (0.41, 2.45) | >0.9 |
| * POOR | 1.17 (0.50, 2.73) | 0.7 | 1.81 (0.70, 4.68) | 0.2 |
